# CXCR4, CXCR7 and PBRM1 are responsible for everolimus and cabozantinib resistance in human renal cancer cells

**DOI:** 10.1038/s41420-026-03026-w

**Published:** 2026-03-28

**Authors:** Federica Auletta, Caterina Ieranò, Dario Guido Di Febbraro, Anna Maria Bello, Giuseppina Rea, Maria Napolitano, Francesca Galdiero, Giuseppe Guardascione, Anna Maria Trotta, Sara Santagata, Cinzia Vetrei, Gaetana Di Maiolo, Daniela Russo, Anna Spina, Crescenzo D’Alterio, Luigi Portella, Serena Ascrizzi, Giovanni Luca Scaglione, Pierfrancesco Tassone, Maria Teresa Di Martino, Stefania Scala

**Affiliations:** 1 Microenvironment Molecular Targets Unit, Istituto Nazionale Tumori - IRCCS –Fondazione G. Pascale, Napoli, Italy; 2 Department of Experimental and Clinical Medicine (DMSC), Magna Graecia University, Catanzaro, Italy; 3 Bioinformatics Unit, Istituto Dermopatico dell’Immacolata IDI-IRCCS, Rome, Italy

**Keywords:** Renal cell carcinoma, Cancer therapeutic resistance

## Abstract

The mTOR inhibitor everolimus (RAD001), previously used in first-line treatment of metastatic renal cancer (mRCC), is currently reserved for the following lines of therapy. However, many patients eventually develop resistance to RAD001. To shed new light on the mechanism of RAD001 resistance, A498 cells resistant to 1–5–10 µM of RAD001 were developed (A498-RAD 1-5-10). A498-RAD-resistant cells overexpressed the chromatin remodeling factor PBRM1 and mTOR and downregulated the chemokine receptors CXCR4 and CXCR7. To reverse RAD001 resistance, PBRM1 knockdown was conducted in A498-RAD10 cells. PBRM1 knockdown partially restored sensitivity to RAD001 while inducing CXCR7 but not CXCR4 expression. In A498-RAD10 cells, the CXCR7 transcriptional repressor YY1 is overexpressed and bound to the CXCR7 promoter. As CXCR4 was robustly downregulated in A498-RAD10, and the PBRM1 knockdown only partially restored RAD001 sensitivity, CXCR4 was transfected into A498-RAD10 (A498-RAD10-CXCR4). CXCR4 completely restored RAD001 sensitivity in resistant cells while reducing PBRM1 expression, implying negative feedback. Interestingly, A498-RAD10 cells were cross-resistant to cabozantinib, the tyrosine kinase inhibitor used in first-line treatment of mRCC with nivolumab. Cabozantinib resistance of A498-RAD10 cells was reversed by PBRM1 knockdown or CXCR4 re-expression, mimicking the RAD001 resistance. A498-CABO4-resistant cells were developed and showed PBRM1 overexpression and downregulated CXCR4 and CXCR7. In silico data supported a context‑dependent role of PBRM1 in ccRCC patients. To the best of our knowledge, this is the first description of a mechanism of RAD001 and cabozantinib resistance through PBRM1 overexpression and CXCR7/CXCR4 downregulation and suggest new therapeutic perspective for cabozantinib-resistant patients.

## Introduction

Renal cell carcinoma (RCC) is the most prevalent form of kidney cancer, with 70% clear cell subtype (ccRCC) [1]. In ccRCC, von Hippel-Lindau (VHL) tumor suppressor gene inactivation stabilizes and accumulates hypoxia factor 1-alpha (HIF1-α) in normoxic conditions. HIF1-α promotes transcription of VEGF, PDGF, TGF-β, EGFR, and CXCR4, inducing neoangiogenesis, cell growth and migration [2, 3]. VHL inactivation alone is insufficient for RCC tumorigenesis, either in VHL-sporadic or -hereditary ccRCC, while co-deletion of VHL plus at least one of three genes encoding proteins involved in chromatin-modifying proteins, polybromo 1 (PBRM1), BRCA1-related protein 1 (BAP1) and SET domain-containing 2 (SETD2), is sufficient for renal cell transformation. These genes are located in proximity to VHL on chromosome 3(3p) [4]. PBRM1 gene coding for the BAF180 protein, a subunit of the PBAF subtype, component of the SWItch/Sucrose Non-Fermentable (SWI/SNF) chromatin remodeling complex [5]. BAF180 includes six bromodomains, two bromo-adjacent homology (BAH) domains, and a high mobility group (HMG) domain [6]. PBRM1 loss-of-function mutation is reported in 40-50% of ccRCC [7], while SWI/SNF loss-of-function mutations are reported in ~20% of human tumors [8]. Clinically, 30% of RCC presents with metastasis and 30% develops advanced/metastatic disease (mRCC). First-line treatments rely on immune checkpoint blockade (ICB)-based regimens, either dual ICBs such as nivolumab (anti-PD-1) plus ipilimumab (anti-CTLA-4) or combinations of ICBs with tyrosine kinase inhibitors (TKIs) such as axitinib, lenvatinib, sunitinib, pazopanib, and cabozantinib, which act on VEGFR, PDGFR, FGFR, AXL and/or c-MET. These combinations have improved patient outcomes compared to previous VEGF-targeted monotherapies [9]. Cabozantinib is a kinase inhibitor targeting multiple kinases VEGFR, MET, RET, KIT and TAM (TYRO3, AXL, MER) [10, 11]. Cabozantinib receptors MER and TYRO3 are expressed on macrophages, dendritic cells, and natural killer cells, suppressing pro-inflammatory cytokines such as IL-6 and TNF, promoting an immunosuppressive environment [12]. Everolimus (RAD001) is a selective mTOR1 inhibitor previously used for the treatment of mRCC patients after failure of VEGFR-TKIs. Today, RAD001 is suggested in the second-line treatment in combination with the TKI agent, lenvatinib [13]. Although the mTOR pathway is functional in RCC patients [14], the majority of patients treated with RAD001 develop resistance with disease progression. The reduced therapeutic efficacy is mainly attributable to the complex interaction between the PI3K/AKT/mTOR and signaling pathways involved in cell growth and proliferation, such as the CXCR4-CXCL12-CXCR7 axis. Previous evidence demonstrates that CXCR4 signal on mTOR in gastric cancer [15], pancreatic cancer [16] and renal cancer [17]. Thus, we investigated the mechanism of RAD001 resistance and the possible role of CXCR4–CXCL12–CXCR7 axis in modulating RAD001 sensitivity in ccRCC.

## Results

### A498 RAD001-resistant cells overexpressed PBRM1/mTOR and downregulated CXCR4/CXCR7

**Fig. 1:**
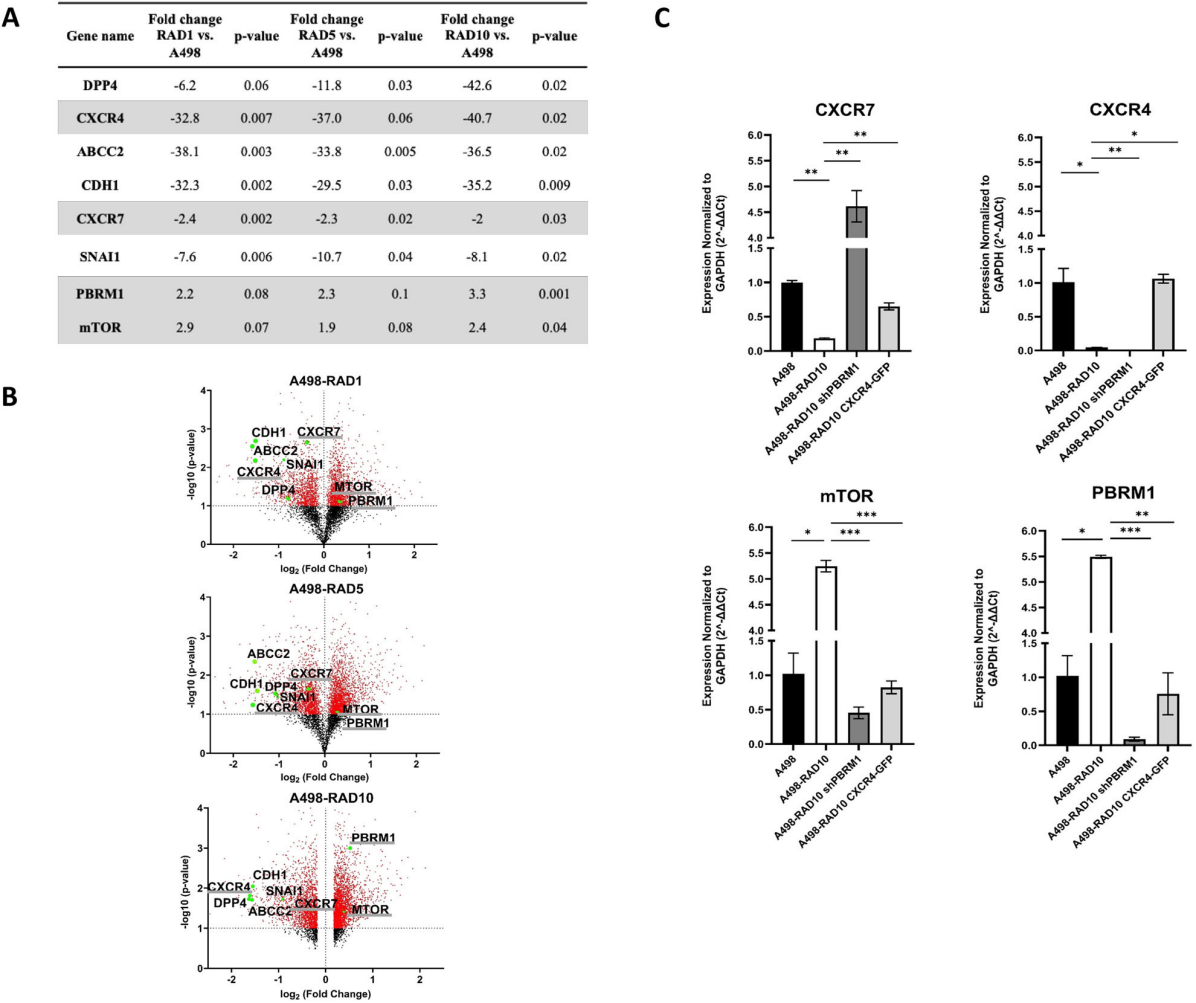
A498 RAD-resistant cells overexpressed PBRM1, mTOR and downregulated CXCR4 and CXCR7. **A** Differentially expressed genes in A498 RAD 1-5-10/ A498. **B** Volcano plot for genes differentially expressed in A498 RAD1-5-10 vs A498 cells. The log_2_ fold change difference is represented on the x-axis and –log_10_ of corrective *p* value is represented on the y-axis. Each point represents a gene. Red points indicate genes that were upregulated (log_2_ fc > 0) or downregulated (log_2_ fc < 0) in A498 RAD1-5-10 vs A498 cells. The genes of interest are underlined. **C** CXCR4, CXCR7, mTOR and PBRM1 expression in A498, A498-RAD10, A498-RAD10 shPBRM1 and A498-RAD10 CXCR4-GFP through qRT-PCR. Data are representative of at least two experiments. Statistical significances were calculated by Student’s *t* test. *P < 0.05, **P < 0.01, ***P < 0.001.

To identify genes crucial for RAD001 resistance, A498-RAD001 resistant cells (1–5–10 µM) were developed (Table S1) and differentially expressed genes were evaluated. CXCR4 and CXCR7 were significantly reduced in A498-RAD 1-5-10 cells (32.8, 37, 40.7-fold for CXCR4 and 2.4, 2.3, 2-fold for CXCR7), while PBRM1 and mTOR were significantly upregulated (2.2, 2.3, 3.3-fold for PBRM1 and 2.9, 1.9, 2.4-fold for mTOR) (Fig. 1A, B). To define the role of PBRM1 in RAD001 resistance, PBRM1 was stably knocked down in A498-RAD10 (A498-RAD10 shPBRM1) (Fig. S1). A498-RAD10 shPBRM1 cells were 2.4-fold more sensitive to RAD001 than A498-RAD10 but 7.1-fold more resistant than A498 (Table 1). This evidence was confirmed by PBRM1 CRISPR/Cas9 in A498-RAD10 (Fig. S2 and Table S2). A498-RAD10 shPBRM1 robustly overexpressed CXCR7 (25.1-fold compared to A498-RAD10) while CXCR4 was unaffected (Fig. 1C). mTOR was upregulated in A498-RAD10 (5.1-fold) and reduced in A498-RAD10 shPBRM1 compared to A498-RAD10 (11.5-fold), suggesting a role for PBRM1 in regulating mTOR expression and, consequently, in the mechanism of RAD001 sensitization (Fig. 1C). Since PBRM1 downregulation only partially restored RAD001 sensitivity in A498-RAD10, other mechanisms should be involved in RAD001 resistance. As shown in Fig. 1C, CXCR4 expression dramatically decreased in the A498-RAD10 (22.3-fold) and even more in A498-RAD10 shPBRM1, suggesting that CXCR4 plays a role in RAD001 resistance PBRM1 unrelated. Thus, CXCR4 was transfected into resistant A498-RAD10 cells (A498-RAD10-CXCR4) (Fig. S3). CXCR4 overexpression completely restored RAD001 sensitivity in A498-RAD10 cells (Table 1). Moreover, A498-RAD10-CXCR4 displayed a dramatic reduction in PBRM1 and mTOR as compared to A498-RAD10 (Fig. 1C). To test the CXCR4/CXCR7–PBRM1 axis in a distinct genetic background, the human RCC line SN12C and RAD001‑resistant derivative, SN12C‑RAD20 [17] were analyzed. A498 is a prototypical clear cell renal carcinoma cell line with biallelic VHL inactivation, canonical HIF‑2α‑driven transcriptional program and molecular features that closely mirror classical ccRCC biology. SN12C is VHL‑proficient with a more heterogeneous, mixed/outlier RCC background, tumour‑initiating, pro‑angiogenic and EMT‑related properties different from typical ccRCC models [18, 19]. SN12C-RAD20 expresses high CXCR7/CXCR4 and low PBRM1, YY1, mTOR and FOXP3 (Fig. S4A) in contrast to the pattern observed in A498‑RAD10, probably due to the genetic differences between cell lines, but confirming the involvement of the axis CXCR4-CXCR7-PBRM-1 in RAD001 resistance.

**Table 1:** IC_50_ ± SD values of A498, A498-RAD10, A498-RAD10shPBRM1, A498-RAD10-CXCR4 for RAD001 and cabozantinib.

IC_50_ (µM)	RAD001	RR	Cabozantinib	RR
**A498**	1.5 ± 1	1	3.8 ± 1.1	1
**A498-RAD10**	25.8 ± 3	17.2	8.3 ± 1.2	2.2
**A498-RAD10 shPBRM1**	10.7 ± 3	7.1	3.6 ± 1.3	1
**A498-RAD10 CXCR4**	1.8 ± 0,8	1	3.1 ± 0.6	1

Data are means of at least two experiments. RR (relative resistance) was calculated as Resistant-Cell IC_50_ / Parental-cell IC_50._

Moreover, the PBRM1 function is mostly recognized as context‑dependent. A498 cells belong to the H2‑type ccRCC subgroup, characterized by predominant HIF‑2α activity, expressing HIF‑2α target genes such as VEGFA and Cyclin D1, together with elevated PBRM1 and mTOR levels [20]. In fact, A498-RAD10 expresses high HIF‑2α‑ targets, VEGFA and Cyclin D1 [3] in addition to PBRM1 and mTOR and in A498-RAD10 shPBRM1 cells, the expression of HIF-2α targets and mTOR decreased, partially restoring sensitivity to RAD001 (Fig. S4B). Thus, in A498, PBRM1, instead of performing as a classical tumor suppressor, promotes tumor growth and drug resistance through HIF-2α-regulated genes such as Cyclin D1 and VEGFA (Fig. S4B).

### PBRM1 negatively regulated CXCR7 expression through YY1

**Fig. 2:**
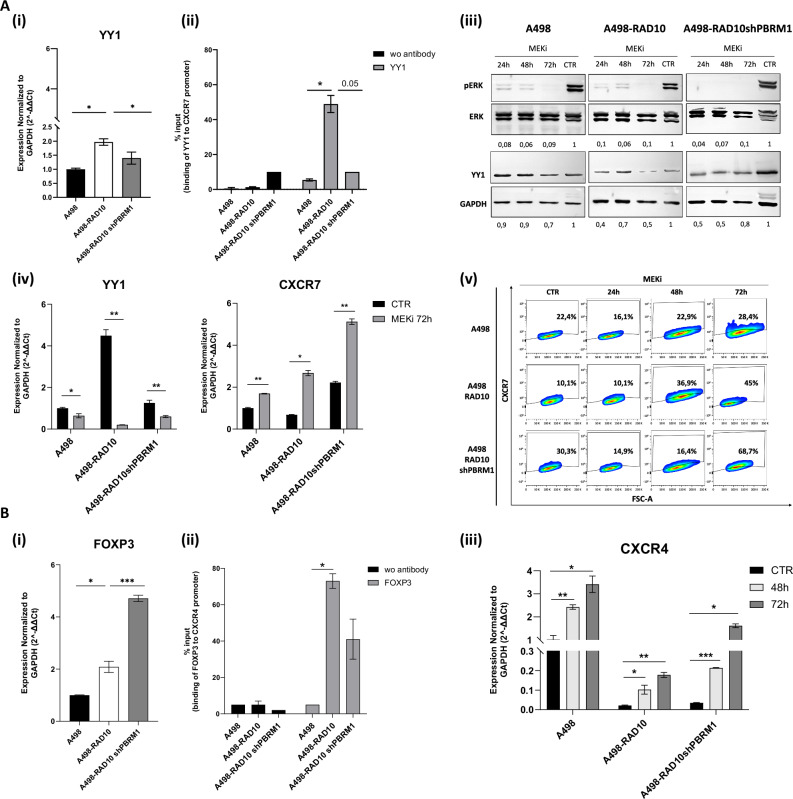
PBRM1 negatively regulated CXCR7 expression through YY1. **A** Analysis of YY1 and CXCR7 regulation: (i) YY1 expression in A498, A498‑RAD10 and A498‑RAD10 shPBRM1 cells measured by qRT‑PCR; (ii) ChIP analysis of YY1 binding to the CXCR7 promoter in A498, A498‑RAD10 and A498‑RAD10 shPBRM1 cells, normalized to input; (iii) western blot analysis of pERK, total ERK and YY1 in cells treated with MEK1/2 inhibitor III (5 µM); (iv) YY1 and CXCR7 expression after MEK inhibition; (v) Flow cytometry CXCR7 surface expression following MEK inhibition. **B** FOXP3–CXCR4 axis: (i) FOXP3 expression in A498, A498‑RAD10 and A498‑RAD10 shPBRM1 cells by qRT‑PCR; (ii) ChIP analysis of FOXP3 binding to the CXCR4 promoter in A498, A498‑RAD10 and A498‑RAD10 shPBRM1 cells, normalized to input; (iii) CXCR4 expression in A498, A498‑RAD10 and A498‑RAD10 shPBRM1 cells treated for 48 and 72 h with the FOXP3‑inhibitory peptide P60 (50 µM). Data are representative of at least two independent experiments. Statistical significance was assessed by Student’s *t* test (*P < 0.05, **P < 0.01, ***P < 0.001).

As CXCR4 and CXCR7 expression dramatically decreased in the A498-RAD10, the expression of the transcription factors FOXP3 and YY1, respectively, CXCR4 and CXCR7 negative regulators, was evaluated. As shown in Fig. 2A(i), YY1 was upregulated in A498-RAD10 cells (~2-fold vs A498) and subsequently decreased in A498-RAD10 shPBRM1 cells (1.4-fold vs A498-RAD10), suggesting that PBRM1 may participate in regulating CXCR7 expression, promoting YY1 transcription by remodeling chromatin accessibility in RAD-resistant cells.

Consistently, in Fig. 2A(ii), chromatin immunoprecipitation (ChIP) showed that YY1 binding was higher on CXCR7 promoter in A498-RAD10 but lower in A498-RAD10 shPBRM1 confirming that PBRM1 regulates YY1 interaction on CXCR7 promoter. To further dissect the mechanistic link between YY1 and CXCR7, YY1 was reduced targeting the RTK–MAPK/ERK axis [21]. A498, A498‑RAD10 and A498‑RAD10 shPBRM1 cells were treated with the MEK inhibitor (MEK1/2 inhibitor III). As shown in Fig. 2A(iii) (Uncropped western blots A), MEK inhibition reduced pERK and YY1 protein with A498‑RAD10 showing the lowest YY1 levels according to higher basal YY1 expression. In Fig. 2A(iv), MEK inhibition significantly reduced YY1 expression and associated with a concomitant increase in CXCR7 expression, particularly in A498-RAD10 and A498-RAD10 shPBRM1 cells. Consistently, in Fig. 2A(v), higher fraction of CXCR7-positive cells was detected particularly in A498‑RAD10 and A498‑RAD10 shPBRM1 cells following MEK inhibition.

FOXP3 was evaluated as a regulator of CXCR4. FOXP3 expression was upregulated in A498-RAD10 cells (~2-fold vs A498) and further increased in A498-RAD10 shPBRM1 cells (2.3-fold vs A498-RAD10), indicating that FOXP3 upregulation occurs independently of PBRM1 status (Fig. 2B(i)). FOXP3 binding on CXCR4 promoter was significantly higher in A498-RAD10 but minimally affected by PBRM1 knockdown (Fig. 2B(ii)), showing that CXCR4 expression is regulated through FOXP3 independently of PBRM1. To further validate this hypothesis, we employed the FOXP3-specific inhibitory peptide P60, which blocks FOXP3 nuclear translocation and transcriptional activity [22]. P60 induced a time-dependent increase in CXCR4 expression regardless of PBRM1 status (Fig. 2B(iii)).

Taken together, these findings show FOXP3 enrichment at the CXCR4 promoter and provide evidence that FOXP3 directly modulates CXCR4 transcription in a PBRM1‑independent manner. YY1, under the control of the MEK/ERK pathway and PBRM1, acts as a key repressor of CXCR7 in RAD001‑resistant A498 cells.

### PBRM1 knockdown in RAD001-resistant cells restores CXCL12/CXCL11 sensitivity through CXCR7

**Fig. 3:**
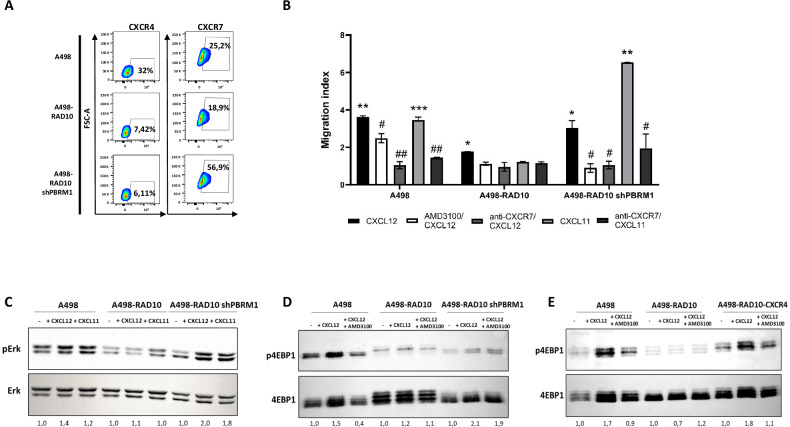
PBRM1 silencing and CXCR4 overexpression in RAD001-resistant cells restores chemokine sensitivity. **A** CXCR4 and CXCR7 level through flow cytometry. Data are representative of at least two experiments. **B** CXCL12/CXCL11-dependent cell migration in A498, A498-RAD10 and A498-RAD10 shPBRM1 in 24-well plates. Cells were placed in the upper chamber (8 µm) in the presence of AMD3100 (1 μM) or anti-CXCR7 (10 μg/ml, 11G8). Cells migrated toward CXCL12 (100 ng/ml), CXCL11 (100 ng/ml) for 18 h. The cells were counted in 10 different consecutive high-power fields (magnification 40x). Each column represents the mean ± S.D. (*n* = 2). Statistical significances were calculated by Student’s *t* test.*P < 0.05, **P < 0.01, CXCL12/ CXCL11 vs CTR; # *P* < 0.05, ##*P* < 0.01, AMD/anti-CXCR7 vs CXCL12. **C** Immunoblot for pERK in A498, A498-RAD10 and A498-RAD10 shPBRM1 treated with CXCL12 or CXCL11 (100 ng/ml for 10 min). **D** Immunoblot for p4EBP1 in A498, A498-RAD10 and A498-RAD10 shPBRM1 treated with CXCL12 or CXCL12 + AMD3100. **E** p4EBP1 immunoblot in A498, A498-RAD10 and A498-RAD10 CXCR4-GFP treated with CXCL12 or CXCL12 + AMD3100. Representative images and the relative quantifications are shown.

CXCR4 level decreased in A498-RAD10 and was unaffected in A498-RAD10 shPBRM1, while CXCR7 decreased in A498-RAD10 but increased in A498-RAD10 shPBRM1 (Fig. 3A). To investigate the function, A498, A498-RAD10 and A498-RAD10 shPBRM1 migration was conducted toward CXCL12, a common ligand for CXCR4 and CXCR7, and CXCL11, a specific CXCR7 ligand. A498 efficiently migrated toward CXCL12 and CXCL11, and migration was impaired by AMD31000, CXCR4 specific inhibitor and by anti-CXCR7. A498-RAD10 migrated less towards CXCL12/CXCL11 with negligible effect of inhibitors, while A498-RAD10 shPBRM1 cells migrated significantly towards CXCL11 and anti-CXCR7 impaired it, confirming the role of PBRM1 in regulating CXCR7 function (Fig. 3B).

**Fig. 4:**
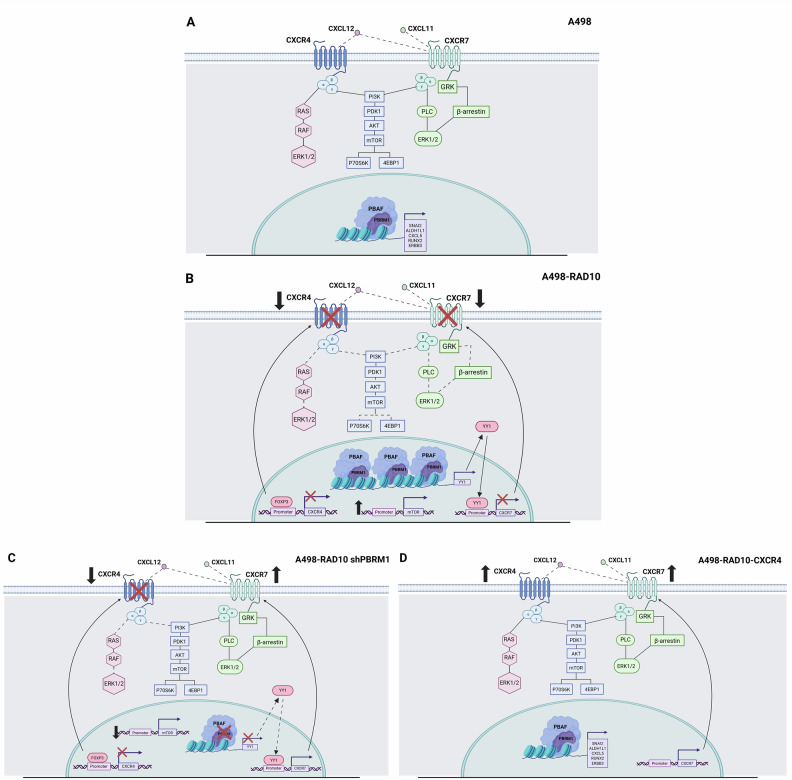
Schematic of RAD001 resistance mechanism and possible interferences. **A** In A498 human renal cancer cells, CXCR4-CXCL12-CXCR7 axis signals on mTOR and ERK. In the nucleus, PBRM1 acts as a chromatin remodeling factor, regulating the expression of several genes [38]. **B** In A498-RAD10 cells, CXCR4 and CXCR7 signaling is impaired due to increased expression of PBRM1 that affects YY1 interaction on CXCR7 promoter reducing the CXCR7 transcription. Moreover, CXCR4 transcription is impaired by FOXP3. **C** In A498-RAD10-shPBRM1, PBRM1 knockdown reduced the YY1 on CXCR7, inducing CXCR7 transcription. FOXP3 interaction on the CXCR4 promoter is unaffected by PBRM1 knockdown. Accordingly, CXCR4 signaling was impaired while CXCR7 signaling on ERK and mTOR was restored. **D** In A498-RAD10-CXCR4, CXCR4 overexpression restored CXCR4 signaling on mTOR and induced the expression of CXCR7.

In addition, CXCL12/CXCL11 induced p-Erk in A498-RAD10 shPBRM1 but not in A498-RAD10 (Fig. 3C-Uncropped western blot B). CXCL12 also induced p4EBP1 in A498-RAD10 shPBRM1, and the CXCL12 induction was unaffected by AMD3100, suggesting that CXCL12 activates CXCR7 in A498-RAD10 shPBRM1 (Fig. 3D- Uncropped Western blot C). Thus, A498-RAD10 cells displayed high PBRM1, low CXCR4 and CXCR7, with PBRM1 knockdown restoring the CXCL12/CXCL11 response through CXCR7. The CXCR4–CXCL12–mTOR axis was also evaluated in A498-RAD10-CXCR4. CXCL12 induced strong 4EBP1 phosphorylation in both A498 and A498-RAD10-CXCR4 cells, whereas AMD3100 completely blocked this effect, confirming CXCR4 drives mTOR/4EBP1 signaling (Fig. 3E- Uncropped western blot D). The schematic of the RAD001 resistance mechanism is recapitulated in Fig. 4.

### A498-RAD10 cells are cross-resistant to cabozantinib

**Fig. 5:**
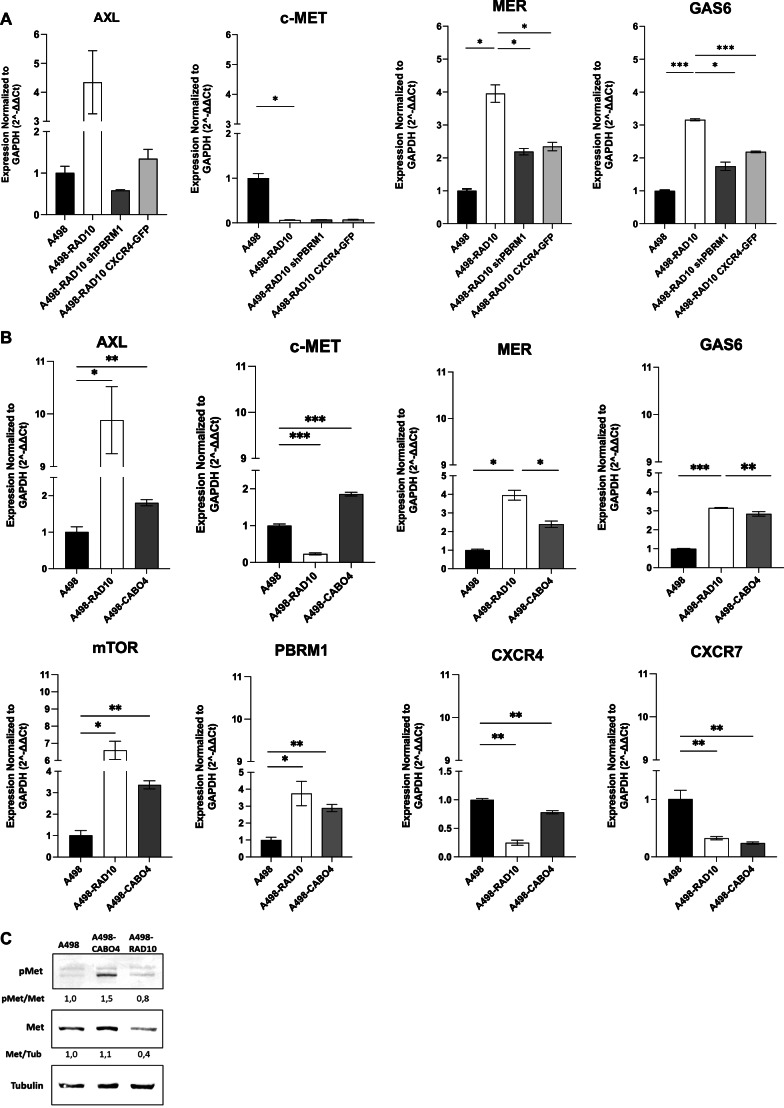
A498-RAD10 cells are cross-resistant to cabozantinib. **A** AXL, c-MET, MER and GAS6 expression in A498, A498-RAD10, A498-RAD10 shPBRM1 and A498-RAD10-CXCR4 and **B** AXL, c-MET, MER, GAS6 mTOR, PBRM1, CXCR4 and CXCR7 expression in A498, A498-RAD10 and A498 CABO4 through qRT-PCR. **C** Immunoblot for pMet in A498, A498-CABO4 and A498-RAD10 cell lines. Representative images and the relative quantifications are shown. Data are representative of at least two experiments. Statistical significances were calculated by Student’s *t* test. *P < 0.05, **P < 0.01, ***P < 0.001.

As TKIs represent a pillar in mRCC treatment, we asked if RAD001 resistance might affect TKIs sensitivity. A498-RAD10 cells were cross-resistant to cabozantinib while A498-RAD10 shPBRM1 and A498-RAD10-CXCR4 were sensitive, suggesting common mechanisms of resistance between RAD001 and cabozantinib (Table 1). No cross-resistance to sunitinib, lenvatinib and axitinib was detected (Table S3). AXL and MER, cabozantinib targets, were significantly upregulated in A498-RAD10 (4.3- and 3.9-fold, respectively) and down-regulated in A498-RAD10 shPBRM1 and in A498-RAD10-CXCR4 compared to A498-RAD10 (Fig. 5A). The other cabozantinib target, c-MET, was dramatically down-regulated in A498-RAD10 (5-fold) and in A498-RAD10 shPBRM1/A498-RAD10-CXCR4, suggesting that this pathway is not involved in the cross-resistance to cabozantinib. Moreover, the growth arrest-specific protein 6 (GAS6), AXL natural ligand, was upregulated in A498-RAD10 (3.2-fold) and down-regulated in A498-RAD10 shPBRM1 and in A498-RAD10-CXCR4 compared to A498-RAD10 (1.8- and 1.4-fold, respectively) (Fig. 5A). To compare the RAD001 and cabozantinib resistance mechanism, A498 cabozantinib-resistant cells were developed (A498-CABO4). A498-CABO4 were 7- and 3.7-fold more resistant, respectively, to RAD001 and cabozantinib (Table S4).

A498-CABO4 cells overexpressed AXL, c-MET, MER and GAS6 (respectively, 1.8-, 1.9-, 2.4- and 2.8 -fold compared to A498) (Fig. 5B). Moreover, as in A498-RAD10, A498-CABO4 overexpressed mTOR (3.4-fold) and PBRM1 (2.9-fold), and expressed lower CXCR4 (1.3-fold) and CXCR7 (4.1-fold) as compared to A498 (Fig. 5B). In Fig. 5C, A498-CABO4 display activated phospho-MET that is absent in A498-RAD10 confirming that MET is not involved in the cross resistance to RAD001 (Fig. 5C- Uncropped western blot E).

To further investigate this difference, MET and AXL signaling were examined in A498 and A498-RAD10 cells following RAD001 treatment. RAD001 binds FKBP12, the binding partner for immunosuppressive drugs FK506 and rapamycin, and forms a complex that disrupts the FKBP12/MET interaction, leading to MET inactivation in sensitive cancer cells like ccRCC lines [23]. In parental A498 cells, RAD001 treatment resulted in a progressive decrease in phospho-MET levels. In contrast, phospho-MET was low and unaffected in A498-RAD10 cells, consistent with a reduced role of MET signaling in the resistant context (Fig. S5).

### In silico analysis of CXCR4, CXCR7, PBRM1, mTOR, YY1 and FOXP3

**Fig. 6:**
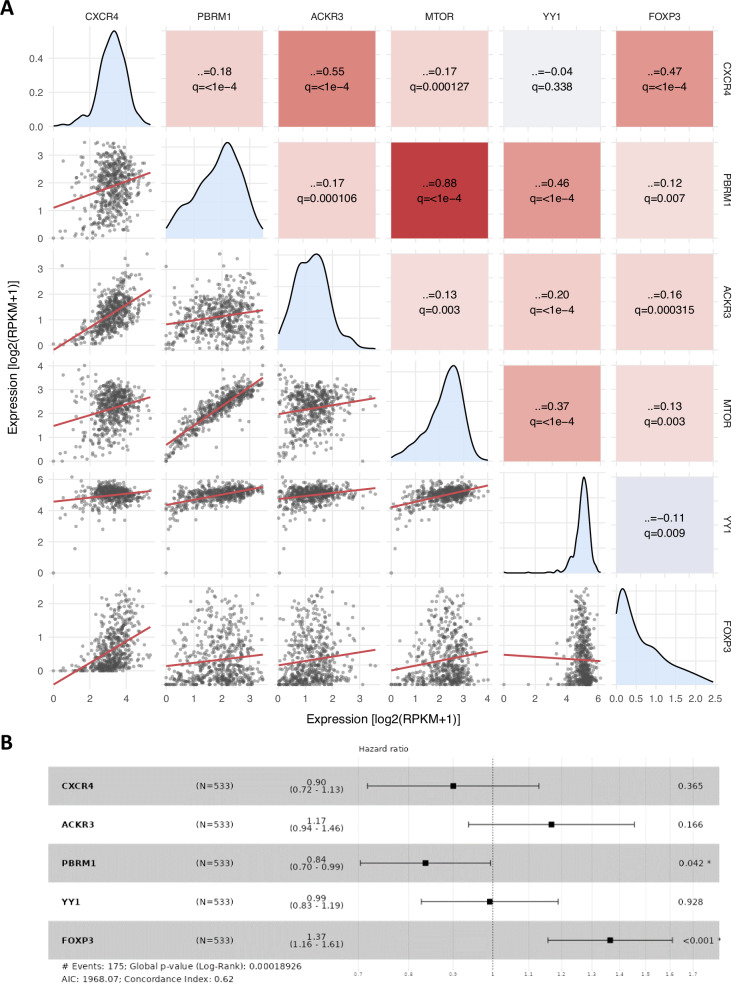
In silico analysis of CXCR4, CXCR7, PBRM1, mTOR, YY1 and FOXP3. **A** Pairwise co‑expression matrix of CXCR4, PBRM1, ACKR3 (CXCR7), MTOR, YY1 and FOXP3 in the TCGA‑KIRC cohort (*n* = 553 primary clear cell renal carcinomas). The matrix reports Spearman’s correlation coefficients (ρ) with Benjamini–Hochberg–adjusted q‑values; lower panels show sample‑wise scatter plots with fitted regression lines, and diagonal panels display kernel density distributions of log2(RPKM + 1) expression values. **B** Forest plot of multivariable Cox proportional hazards analysis for CXCR4, ACKR3 (CXCR7), PBRM1, YY1 and FOXP3 in TCGA‑KIRC patients, showing hazard ratios with 95% confidence intervals for overall survival.

The TCGA‑KIRC cohort (*n* = 553 primary clear cell renal carcinomas) was interrogated for CXCR4, CXCR7, PBRM1, mTOR, YY1 and FOXP3 expression. Spearman’s correlation analysis revealed significant positive associations between CXCR4 and CXCR7 (*r* = 0.55, *p* < 0.0001), PBRM1 and YY1 (*r* = 0.46, *p* < 0.0001), PBRM1 and mTOR (*r* = 0.88, *p* < 0.0001), CXCR4 and FOXP3 (*r* = 0.47, *p* < 0.0001) and YY1 and mTOR (*r* = 0.37, *p* < 0.0001) (Fig. 6A). Multivariable Cox proportional hazards model was used to evaluate the association of PBRM1, ACKR3 (CXCR7), CXCR4, FOXP3 and YY1 with overall survival. High PBRM1 expression was associated with better prognosis (*p* = 0.042), while high FOXP3 expression correlated with worse overall survival (*p* < 0.001). No significant prognostic correlations with YY1, CXCR7 and CXCR4 (Fig. 6B). These data indicate that PBRM1 predominantly behaves as a protective factor in ccRCC patients, suggesting that the functional impact of PBRM1 may vary according to the biological and genetic context. Kaplan–Meier curves for OS were generated by stratifying patients according to the median log2(count + 1) expression using TCGEX (https://tcgex.iyte.edu.tr/). Patients with low PBRM1, low YY1 or high FOXP3 expression displayed significantly worse OS (log‑rank *p* = 0.0061, *p* = 0.0047 and *p* = 0.00013, respectively), while high CXCR4 and CXCR7 expression showed only a trend toward poor outcome (*p* = 0.24 and *p* = 0.078, respectively), in line with previous reports [24–27] (Figure S6A–F). Because the KIRC dataset includes only five patients treated with RAD001, we next analyzed the impact of PBRM1, CXCR7 and CXCR4 expression on survival in everolimus‑treated patients (*n* = 130) from the CheckMate 025 trial [28, 29], using the median log2(count + 1) value as a cut‑off. Pairwise Spearman correlations in this cohort confirmed the link observed in TCGA‑KIRC for PBRM1–mTOR, CXCR4–CXCR7 and CXCR4–FOXP3 (*r* = 0.33, *p* = 1.2 × 10⁻⁴; *r* = 0.219, *p* = 0.012; and *r* = 0.182, *p* = 0.039, respectively). Kaplan–Meier analysis did not show significant prognostic associations for CXCR4, PBRM1 or CXCR7 in RAD001‑treated patients (log‑rank *p* = 0.5, *p* = 0.15 and *p* = 0.41, respectively). Nevertheless, an inverse trend with poorer survival was observed in patients with high PBRM1 expression (Fig. S7), consistent with the notion that RAD001 resistance, as seen in A498‑RAD10 cells, is associated with elevated PBRM1.

## Discussion

Human renal cancer cells A498-RAD001 resistant displayed significantly low expression of CXCR4/CXCR7 and overexpression of PBRM1/mTOR. PBRM1 knockdown partially restored RAD001 sensitivity, inducing CXCR7 but not CXCR4 expression. In A498-RAD10 shPBRM1, the CXCR7 transcriptional repressor YY1 reduced the binding to the CXCR7 promoter, inducing CXCR7 transcription. Differently, the transcription factor FOXP3 binding on CXCR4 promoter increased in A498-RAD10, according to reduced expression of CXCR4, but was unaffected in A498-RAD10 shPBRM1. Thus, RAD001 acquired resistance required a higher PBRM1 that impairs CXCR7 expression. As PBRM1 inhibition only partially restored RAD001 sensitivity, CXCR4 was transfected into A498-RAD10. CXCR4 transfection completely re-induced RAD001 sensitivity in A498-RAD10. Interestingly, A498-RAD10 was cross-resistant to cabozantinib, and A498-RAD10 shPBRM1 /A498-RAD10-CXCR4 were susceptible to cabozantinib.

PBRM1 is primarily described as a tumor suppressor in ccRCC, as loss-of-function mutations are frequently associated with aggressive disease phenotypes [7, 30]. Mechanistically, PBRM1 acts as a chromatin regulator and a co-activator of two HIF transcription factors with opposing roles in ccRCC: HIF-1α, which exerts tumor-suppressive effects, and HIF-2α, which promotes tumor growth and progression. In cellular contexts lacking HIF-1α expression, PBRM1 has been shown to preferentially support HIF-2α–driven transcriptional programs, thereby enhancing cell survival and proliferation [20]. In this study, the role of PBRM1 in RAD001 resistance emerges as highly context-dependent. In A498 cells, which belong to the H2‑type ccRCC subgroup with predominant HIF‑2α activity, PBRM1 is upregulated in RAD001‑resistant derivatives, sustaining HIF‑2α target genes and mTOR expression, thereby promoting cell survival and drug resistance. By contrast, in the genetically distinct SN12C/SN12C‑RAD20 model, RAD001 resistance is associated with CXCR4/CXCR7 upregulation and PBRM1 downregulation, underlining the context for the PBRM1 role. In ccRCC patients, despite a strong correlation between PBRM1 loss and high risk of relapse and recurrence [31], the predictive value of PBRM1 remains unclear. While in PBRM1 mutated RCC cells RAD001 resistance was reported [32], in relapsed disease, PBRM1 loss improved response to anti-angiogenetic agents, mTOR inhibitors and ICBs [33, 34]. In the IMmotion150 trial, a randomized phase II study comparing sunitinib with atezolizumab versus atezolizumab with bevacizumab as first-line treatment for previously untreated renal cancer patients, PBRM1-mutated patients were less responsive to ICBs [35]. In contrast, ccRCC patients with PBRM1-mutated tumors showed a significant benefit in PFS and OS when treated with nivolumab [36]. Interestingly, A498-RAD10 cells are cross-resistant to the TKI cabozantinib. Cabozantinib is a kinase inhibitor targeting VEGFR, MET, RET, KIT and TAM (TYRO3, AXL, MER) [10, 11], with AXL being predominant in renal cancer (59–73%) but absent in normal kidney [37, 38]. Cabozantinib improved the overall survival compared to RAD001 as second-line treatment of mRCC in patients previously treated with TKIs (METEOR) [39] and offers a significant clinical benefit compared to sunitinib as first-line therapy in patients with mRCC (CABOSUN) [40]. A498-RAD10 cells were cross-resistant to cabozatinib through AXL, MER and GAS6, AXL ligand [30], overexpression. Surprisingly, MET was significantly downregulated in A498-RAD10 cells. Probably this is due to RAD001 impairment of MET phosphorylation through interruption of the MET-FKBP12 complex [21]. Thus, in A498-RAD10 resistant cells, MET activation might be aberrant and thus less responsive to RAD001 inhibition. However, functional studies, including the use of selective AXL inhibitors, will be required to determine whether AXL activation is causally involved in RAD001 resistance.

Within the NIVOREN GETUG-AFU 26 trial, assessing the activity and safety of nivolumab in patients with metastatic ccRCC, AXL-positive tumors associated with worse progression-free survival, while PBRM-1 loss, detected in 15% of the 324 patients, predicted better PFS [41]. AXL, MER and GAS6 upregulation was detected in A498-CABO4 cancer cells. The upregulation of both MET and AXL detected in A498-CABO suggests that resistance to cabozantinib requires reactivation/overexpression of escape pathways targeted by cabozantinib, rather than a compensatory switch from one to the other. CXCR4 and CXCR7 are downregulated in both A498-RAD10 and A498-CABO4. Thus, the cross-resistance between RAD001 and cabozantinib in RCC is complex and relies on the overlapping mechanisms of mTOR, PBRM1, CXCR4, CXCR7, AXL, and c-MET signaling pathways.

In silico analyses further support the notion that PBRM1 exerts a context-dependent role in ccRCC. In the TCGA-KIRC cohort, PBRM1 expression was associated with a favorable prognosis, consistent with tumor-suppressive function. Notably, in the CheckMate025 cohort of RAD001-treated patients, although no statistically significant association was observed for CXCR4, CXCR7 or PBRM1, a trend toward poorer survival was evident in tumors expressing higher PBRM1 levels. This observation mirrors our experimental findings in A498-RAD10 cells, in which elevated PBRM1 contributes to RAD001 resistance, and suggests that, under specific therapeutic and molecular contexts, PBRM1 may shift from a predominantly protective role to one that supports drug resistance.

To the best of our knowledge, this is the first description of a mechanism of RAD001 resistance through PBRM1 overexpression and CXCR7/CXCR4 downregulation. The cross-resistance between RAD001 and cabozantinib potentiates the interest as cabozantinib is largely used in first-line treatment for mRCC patients and may suggest new therapeutic options.

## Materials and methods

### Reagents

RAD001 (cat. 159351-69-6) and AMD3100 (cat. 239820-5MG) were purchased from Sigma-Aldrich (St. Louis, MO, USA). The anti-CXCR7 was obtained from R&D Systems (Minneapolis, MN, USA). Cabozantinib, lenvatinib and axitinib were purchased from MedChemExpress (Monmouth Junction, NJ, USA). MEK1/2 inhibitor III was supplied by Calbiochem (San Diego, CA). Peptide P60 was supplied by MedChemExpress.

### Cell culture and transfection

RAD001-resistant A498 cell lines were selected by exposing parental A498 to 1 μM, or 5 μM, or 10 μM RAD001 over 12 months. Cabozantinib-resistant A498 cells were selected by exposing parental A498 to 4 μM of cabozantinib for 6 months. Cells were cultured in Dulbecco’s Modified Eagle Medium (Sigma) supplemented with 10% fetal bovine serum (FBS) and 1% penicillin and streptomycin (Invitrogen). Lentiviral particles encoding a pool of four shRNAs specific for PBRM1 inhibition (2.5 × 10^4^ IFU; shPBRM1, sc-76075-V, Santa Cruz Biotechnology) were used. 6 × 10^4^ cells were plated in a 24-well plate 24 h prior to viral infection. Cells were incubated with Polybrene at 5 μg/ml and transfected with the viral particles. Control shRNA Lentiviral Particles-A (2.5 × 10^4^ IFU; shControl, sc-108080, Santa Cruz Biotechnology, Dallas, TX, USA) were used. Clones expressing shPBRM1 and the scrambled shRNA were selected with 5 μg/ml of puromycin selection. For CXCR4-GFP lentiviral particles, the CXCR4-pLenti-C-mGFP-P2A-Puro (Origene CAT#: RC202069L4) plasmid was co-transfected with pCMVdR8.2 and pCMV-VSV-G plasmids into HEK293T cells by calcium phosphate. Supernatant containing lentiviral particles was harvested 48- and 72 h post-transfection, pooled together and centrifuged 5 min at 1200 rpm at 4 °C. A498-RAD10 cells were stably infected with lentiviral particles and 8 mg/ml polybrene (Sigma-Aldrich) for 48 h.

### Cytotoxicity assay

Cells were plated in 96-well plates (1000 cells/well), treated with drugs and cytotoxicity effect evaluated with the SRB assay. Cells were treated and incubated at 37 °C with 5% CO_2_ for 5 days. Cell viability was calculated (OD treated cells/OD untreated cells x 100). IC_50_ is the drug concentration that reduces cell number by 50%.

### ImmunobloBlot

Cells were lysed in a whole-cell buffer containing protease and phosphatase (10 mM NaF, 10 mM Na-pyrophosphate, 1 mM Na_3_VO_4_) inhibitors. Rabbit monoclonal antibodies for p44/42 MAPK (ERK1/2) (Cat# 9102, RRID: AB_330744), phospho-p44/42 MAPK (ERK1/2; T202/Y204) (Cat# 9101, RRID: AB_331646), phospho-4EBP1 (T70) (Cat# 9455, RRID: AB_330949), 4EBP1 (53H11) (Cat# 9644, RRID: AB_2097841), pMET (Y1349) (Cat# 3133, RRID: AB_2181539), mouse monoclonal antibody for MET (L41G3) (Cat# 3148, RRID: AB_1031042), YY1 (D5D9Z) rabbit monoclonal antibody (Cat# 46395, RRID: AB_2799302) were obtained from Cell Signaling (Danvers, Massachusetts, USA). Secondary antibody includes goat anti-rabbit-HRP (Jackson ImmunoResearch, West Grove, Pennsylvania, USA) and goat anti-mouse-HRP (Proteintech, Rosemont, Illinois, USA). The signal was revealed through chemoluminescence (Pierce ECL Western Blotting Substrate, Thermo Fisher Scientific). Protein expression was detected with Image Acquisition using iBright Imaging Systems (Invitrogen).

### Flow cytometry

For cell-surface markers, 200.000 cells/tube were incubated with specific or isotype control antibodies in the dark for 30 min at 4 °C in staining buffer. PE anti-hCXCR4 (12G5) (Cat# FAB170P, RRID: AB_357076) and BV421 anti-CXCR7 antibody (10D1) (Cat# 566233, RRID: AB_2744473) were from BD Biosciences (San Jose, California, USA). Isotype control antibodies were used to determine the level of non-specific binding (PE IgG2a, k and BV421 IgG2a, k, BD Biosciences). Stained cells were evaluated with a LSR Fortessa X-20, BD Biosciences and all data were analyzed using FlowJo software (BD Biosciences).

### Migration assay

Migration was performed in 24-well transwell chambers using inserts (8-μm pore-size). Membranes were pre-coated with collagen and fibronectin. Cells were placed in the upper chamber (2 × 105 cells/ well) in DMEM containing 0.5% BSA (migration media). Cells were pre-treated with the CXCR4 antagonist AMD3100 10 μM (Sigma) or anti-CXCR7 10 μg/ml (11G8, R&D Systems) for 30 min and then CXCL12 and CXCL11 100 ng/ml (R&D Systems) were added to the lower chamber. Cells were fixed in 4% paraformaldehyde in PBS 1x and stained for 15 min with DAPI. Migrated cells were visualized (Carlo Zeiss, Axio Scope A1) and counted in 10 different consecutive high-power fields (magnification 40x). Migration is reported as migration index (migrating cells toward CXCL12/number of cells migrating toward BSA 0.5% medium).

### RNA isolation and real-time reverse transcription-polymerase chain reaction

RNA was extracted from cell lines with TRIzol Reagent (Invitrogen, Carlsbad, California, USA) following the manufacturer’s instructions. cDNA was synthesized using 200 ng RNA and 100 U Superscript III (Invitrogen, Karlsruhe, Germany) and random hexamer primers (Invitrogen) according to the manufacturer’s instructions. The gene-specific primers used for the amplification were as follows:

CXCR4 FW: 5’-TGGGTGGTTGTGTTCCAGTTT-3’

RV: 5’-ATGCAATAGCAGGACAGGATGA -3’

CXCR7 FW: 5’-ACAGCACAGCCAGGAAGG-3’

RV: 5’-GTTCTGAGGCGGGCAATC-3’

YY1 FW: 5’- AGTGGGAGCAGAAGCAGG -3’

RV: 5’- TCATGGCCGAGTTATCCC -3’

FOXP3 FW: 5’- AGCACATTCCCAGAGTTCCT -3’

RV: 5’- TGGCGTAGGTGAAAGGGG -3’

mTOR FW: 5’-TGCTGCGTGTCTTCATGCAT-3’

RV: 5’-GGATTGCAGCCAGTAACTTGATAG-3’

AXL FW: 5’-CAGCTTCTCCTTCAGCTCTTCAC-3’

RV: 5’-AACCTTCAACTCCTGCCTTCTCG-3’

c-MET FW: 5’-CTGCCTGCAATCTACAAGGT-3’

RV: 5’-ATGGTCAGCCTTGTCCCTC-3’

MER FW: 5’-GATTGGAGACAGGACCAAAGC-3’

RV: 5’-GACGTAAATAACGTCTGCTTGG-3’

GAS6 FW: 5’-CATCAACAAGTATGGGTCTCCGT-3’

RV: 5’-GTTCTCCTGGCTGCATTCGTTGA-3’

GAPDH FW: 5’- ACCACCATGGAGAAG -3’

RV: 5’- CTCAGTGTAGCCCAG -3’

### Gene expression profiling

100 ng of total RNA from A498 parental and RAD001 resistant cell lines (1–5–10) derived from two independent biological duplicates were expedited through the Gene Chip WT PLUS Reagent Kit (Thermo Fisher Scientific, Waltham, MA, USA) according to the manufacturer’s instructions. Expression profiling was conducted on GeneChip™ Human Gene 1.0 ST Array (Thermo Fisher Scientific, Waltham, MA, USA). The GeneChip TM 3000 system was used for the hybridization, staining, and washing process. Resulting data (CEL files) were produced by Gene Chip Command Console software and further analyzed by Transcriptome Analysis Console (TAC) software v 4.0 (Applied Biosystems, Thermo Fisher, Waltham, MA, USA). Data were normalized by the Signal Space Transformation-Robust Multi-Array Average (SST-RMA) algorithm. Difference of comparative gene expression was identified by the fold change (FC) ≤ − 2.0 or ≥2.0 and *p* value < 0.05. The HTA 2.0 library available on the TAC 4.0 software was used to annotate results.

### Chromatin immunoprecipitation (ChIP) assay

ChIP assay was performed using the ChromataChIP Kit (Novus Biologicals) (cat: NBP1-71709) following the manufacturer’s protocol. Briefly, 80% confluent cells were cross-linked for 10 min in 1% formaldehyde at room temperature with gentle agitation. Cells were lysed post 15-min incubation on ice in RIPA lysis buffer containing protease inhibitors. DNA in the cell lysate was then sheared into 200- to 900-bp fragments by sonication (6 × 15 s impulses/1 min rest on ice between impulses). AhR/chromatin complexes were pulled down by overnight incubation with YY1 (D5D9Z) (Cat# 46395, RRID: AB_2799302) Rabbit monoclonal antibody (Cell Signaling, Danvers, Massachusetts, USA) and anti-hFOXP3 (Cat# AF3240, RRID: AB_2262812) (R&D, Minneapolis, USA), caught by protein A/G magnetic beads. H3K4me3 rabbit polyclonal antibody (NB21-1023) was used as a positive control. DNA was purified by silica columns (ChromataChIP kit) and used for quantitative real-time polymerase chain reactions performed as described below. Sequences of RPL30 primers included in the ChromataChip kit were used as a positive control (Novus Biologicals proprietary). The sequence of primers employed is the following:

FOXP3 FW: 5’- TCCGGGCTTATTTGC-3’

RV: 5’- TCACTAGGGTCAGGT-3’

YY1 FW: 5’-AAAGCCAGAGCATTGCACATGGGA-3’

RV: 5’-TCTGAGATGAGTCTGGCAAACACT-3’

### Bioinformatics analysis

#### Data source

Transcriptome Analysis (TCGA-KIRC): RNA-Seq data were obtained from UCSC Xena (dataset “TCGA.KIRC.sampleMap/lluminaHiSeq_HiSeqV2_exon”, units log2[RPKM + 1]). The analysis was restricted to *n* = 533 primary tumor samples (barcode “01”).

#### Correlation analysis

Pairwise gene–gene associations were evaluated using Spearman’s rank correlation coefficient (ρ or rho), a non-parametric measure robust to non-normal data distributions. For each gene pair, the following metrics were computed: Spearman correlation coefficient (ρ), two-sided p-value, number of evaluable samples, q-value that represents p-value adjusted for the False Discovery Rate (FDR), calculated using the Benjamini–Hochberg (BH) procedure across all tested pairs. Only sample pairs with finite expression values for both genes were included in the analysis. Baseline co-expression among selected targets—CXCR4, ACKR3 (CXCR7), PBRM1, MTOR, YY1, and FOXP3—was evaluated using Spearman’s rank correlation (rho) and displayed by matrix (pairs matrix). This matrix is composed of density plots showing the expression distribution for each gene (color intensity represents the strength of the association), and scatter plots with linear regression lines showing individual sample correlations. The analyses were conducted in R (version ≥4.1) using the packages dplyr, readxl, ggplot2, GGally, ppcor, and openxlsx.

### Statistical analysis

The values given are means ± S.D. Student’s *t* test was used for comparing the means, and differences with a *P*-value of <0.05 were considered significant. The values given are means ± S.D. Student’s *t* test was used for comparing the means, and differences with a *P*-value of <0.05 were considered significant. Variance similarity was verified before parametric testing using the F-test. For each independent experiment, technical replicates were performed at least in triplicate for each data point or drug concentration to ensure measurement precision.

## Supplementary information

Supplementary

Supplementary Materials and Methods

Uncropped Western blot

## Data Availability

The datasets used and/or analysed during the current study are available from the corresponding author on reasonable request. Datasets are available at 10.5281/zenodo.15791745.
