# Supplementary material for: CXCR4, CXCR7 and PBRM1 are responsible for everolimus and cabozantinib resistance in human renal cancer cells
Source: Cell Death Discov. 2026 Mar 28;12:202. doi: 10.1038/s41420-026-03026-w (PMC13149507; doi:10.1038/s41420-026-03026-w)
Supplement: Supplementary file 1 — Supplementary [file 41420_2026_3026_MOESM1_ESM.docx]

| **Cell lines** | **RAD001 IC_50_ (μM)** | **RR** |
| --- | --- | --- |
| A498 | 1.8±0.7 | 1 |
| A498-RAD1 | 10.3±4.2 | **5.7** |
| A498-RAD5 | 12.6±3.1 | **7** |
| A498-RAD10 | 22.3±6.2 | **12.4** |

**Table S1.** IC_50_ value (μM) for RAD001 in A498-RAD1, A498-RAD5 and A498-RAD10 cell lines. Values are indicated as mean ± SD (n=2). RR (relative resistance) is calculated as Resistant-Cell IC50 / Parental-cell IC50.

**
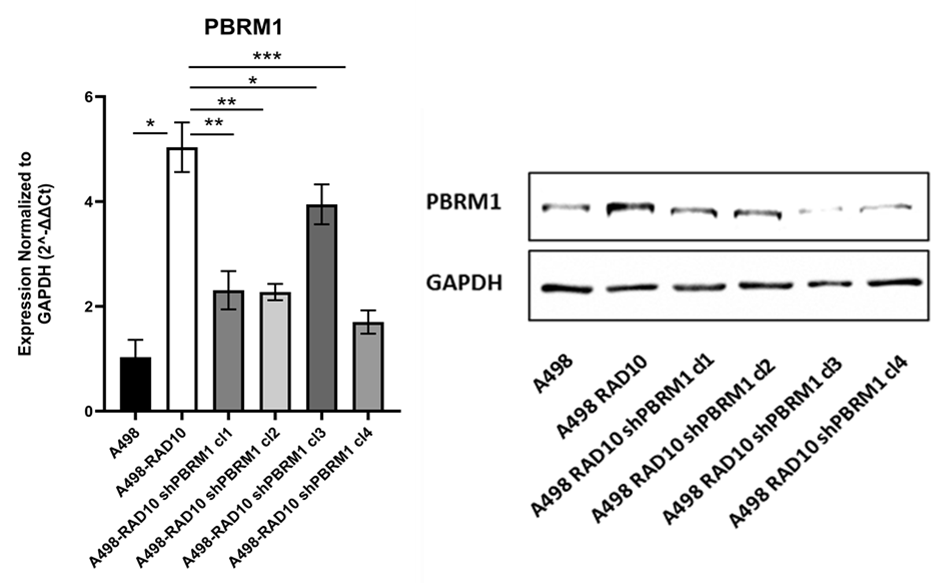
**

**Figure S1.** mRNA relative levels of PBRM1 gene and PBRM1 protein in A498-RAD10 clones stably knocked-down for PBRM1 (A498-RAD10 shPBRM1) compared to A498 and A498-RAD10 cell lines stably expressing a control shRNA. Data are representative of at least two experiments. Statistical significances were calculated by Student’s t-test. *P<0.05,**P<0.01,***P<0.001.


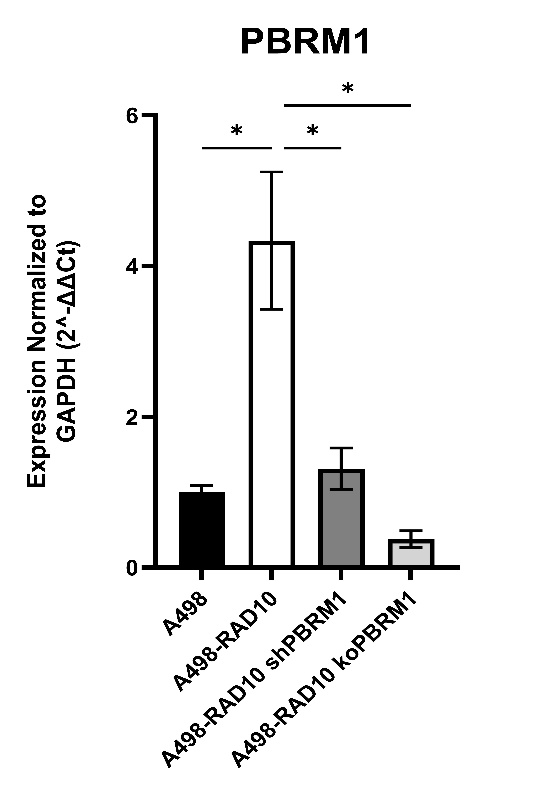


**Figure S2.** mRNA relative levels of PBRM1 gene in A498-RAD10 stably for knocked-out PBRM1 (A498-RAD10 koPBRM1) compared to A498 and A498-RAD10 cell lines. Data are representative of at least two experiments. Statistical significances were calculated by Student’s t-test. *P<0.05

|  | **RAD001 IC_50_ (µM)** | **RR** |
| --- | --- | --- |
| **A498** | 1±0.04 | 1 |
| **A498-RAD10** | 20±0.03 | **20** |
| **A498-RAD10**  **KO PBRM1** | 11±0.08 | **11** |

**Table S2.** IC50 value (μM) for RAD001 in A498, A498-RAD10 and A498-RAD10 koPBRM1 cell lines. Values are indicated as mean± SD (n=2). RR (relative resistance) is calculated as Resistant-Cell IC50 / Parental-cell IC50.

**
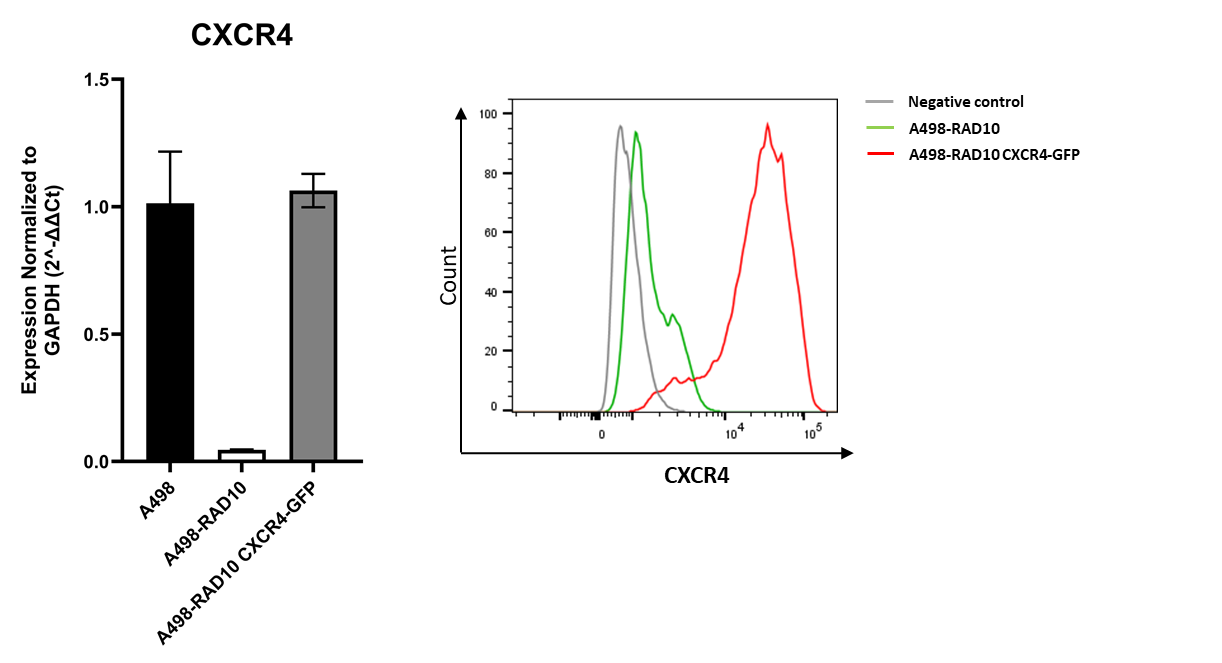
**

**Figure S3.** Transfection of CXCR4-GFP was confirmed through qRT- PCR and flow cytometry analysis in A498, A498-RAD10 and A498-RAD10 CXCR4-GFP. Data are representative of at least two experiments.


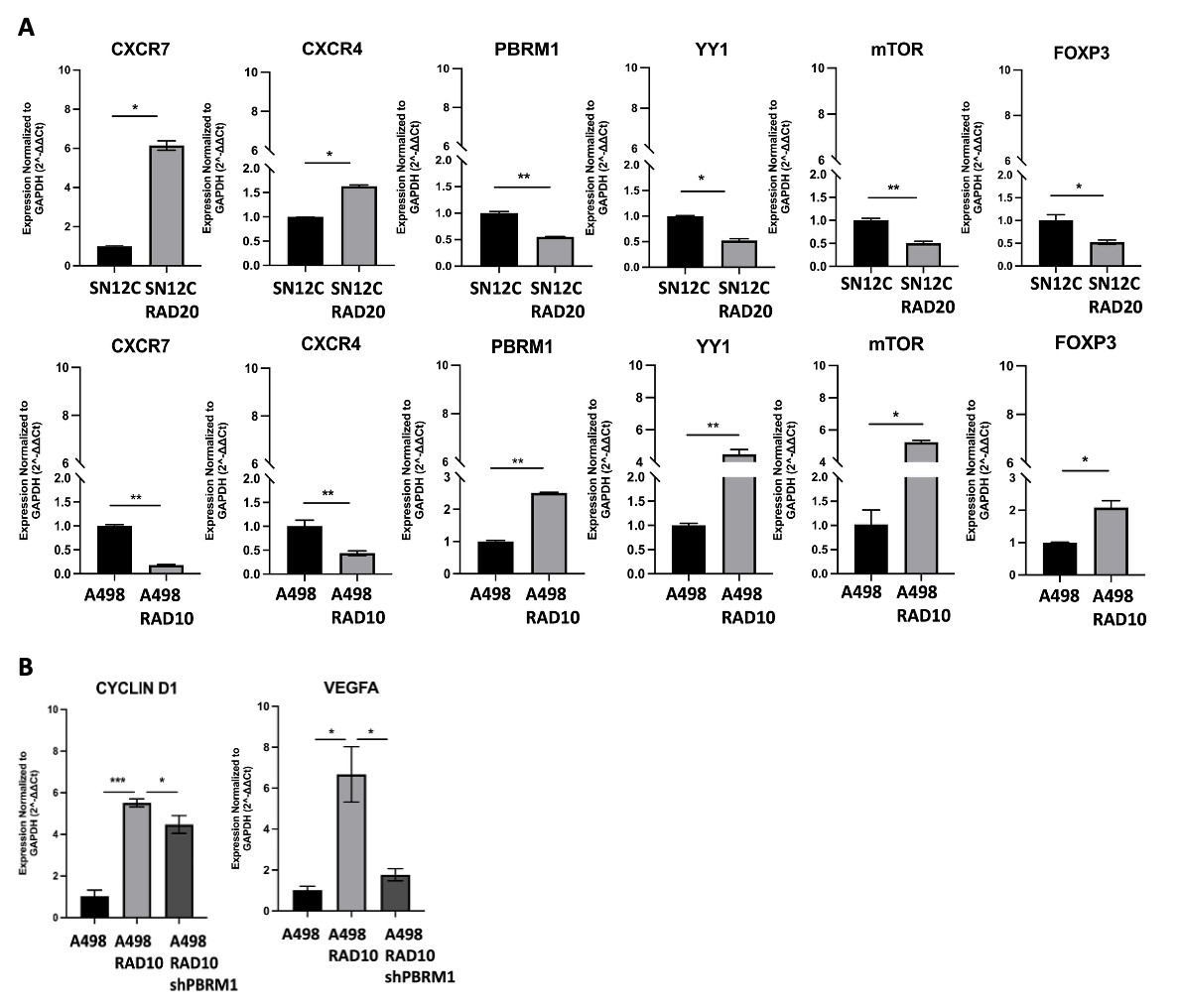


**Figure S4. (A)** Upper panel: high CXCR7, CXCR4 and low PBRM1, YY1, mTOR, and FOXP3 in SN12C-RAD20 compared to SN12C. Lower panel: low CXCR7, CXCR4 and high PBRM1, YY1, mTOR, and FOXP3 in A498-RAD10 compared to A498. **(B)** Cyclin D1 and VEGFA are regulated according to PBRM1 in A498, A498-RAD10 and A498-RAD10shPBRM1 cells.

| **IC_50_ (µM)** | | | | |
| --- | --- | --- | --- | --- |
|  | **A498** | **A498-RAD10** | **A498-RAD10**  **shPBRM1** | **A498-RAD10**  **CXCR4** |
| **Axitinib** | 1.1 ± 0.4 | 1.1 ± 0.2 | 1 | 1.6 ± 0.9 |
| **Lenvatinib** | 19.5 ± 6.4 | 19.5 ± 0.7 | 18.5 ± 5 | 20.5 ± 0.7 |
| **Sunitinib** | 1.4 ± 0.6 | 1.8 ± 0.7 | 2.2 ± 0.6 | 2.5 ± 1 |

**Table S3.** IC50 value (μM) for axitinib, lenvatinib and sunitinib in A498, A498-RAD10 and A498-RAD10shPBRM1 and A498-RAD10-CXCR4 cell lines. Values are indicated as mean± SD (n=2). RR (relative resistance) is calculated as Resistant-Cell IC50 / Parental-cell IC50.

| **IC50 (µM)** | **RAD001** | **RR** | **Cabozantinib** | **RR** |
| --- | --- | --- | --- | --- |
| **A498** | 1.5 ± 1 | 1 | 3.8 ± 1.1 | 1 |
| **A498-RAD10** | 25.8 ± 3 | **17.2** | 8.3 ± 1.2 | **2.2** |
| **A498-CABO4** | 10.5 ± 6 | **7** | 13.5 ± 0.7 | **3.7** |

**Table S4.** IC50 value (μM) for RAD001 and cabozantinib in A498, A498-RAD10 and A498-CABO4 cell lines. Values are indicated as mean± SD (n=2). RR (relative resistance) is calculated as Resistant-Cell IC50 / Parental-cell IC50.

**Figure S5. MET and AXL signaling in A498 and A498-RAD10 cells upon RAD001 treatment.** pMET, MET and total AXL in parental A498 and A498-RAD10 cells treated with RAD001 for the indicated times. RAD001 reduced phospho-MET levels in A498 cells, whereas remained low in A498-RAD10 cells. Total AXL expression appeared increased in A498-RAD10 cells.


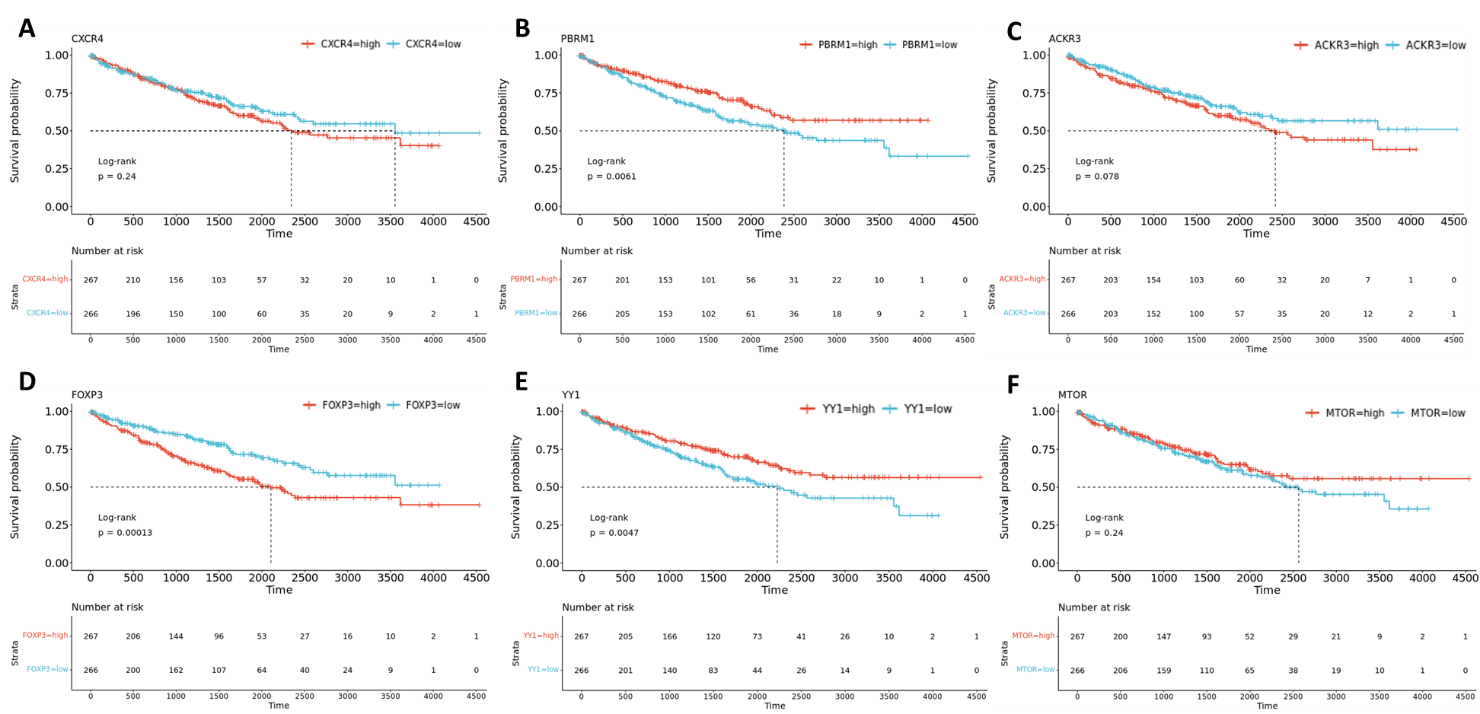


**Figure S6 Prognostic value of PBRM1, CXCR4, CXCR7, FOXP3, YY1 and MTOR in TCGA-KIRC. Overall survival (OS) analyses were performed within KIRC-TCGA cohort to explore the clinical relevance of gene expression patterns classifying samples as high or low expression relative to the gene-specific median.** Kaplan-Meier OS curves for patients stratified as high and low according to median expression of (A) PBRM1, (B) CXCR4, (C) CXCR7, (D) FOXP3, (E) YY1 and (F) MTOR. P-values are from the log-rank test. (A-F). Kaplan–Meier survival curves were estimated for each comparison and evaluated using the log-rank test. Univariable Cox proportional hazards regression models were fitted to estimate hazard ratios (HRs) and 95% confidence intervals. Only comparisons with complete survival information and a minimum number of evaluable samples were considered.


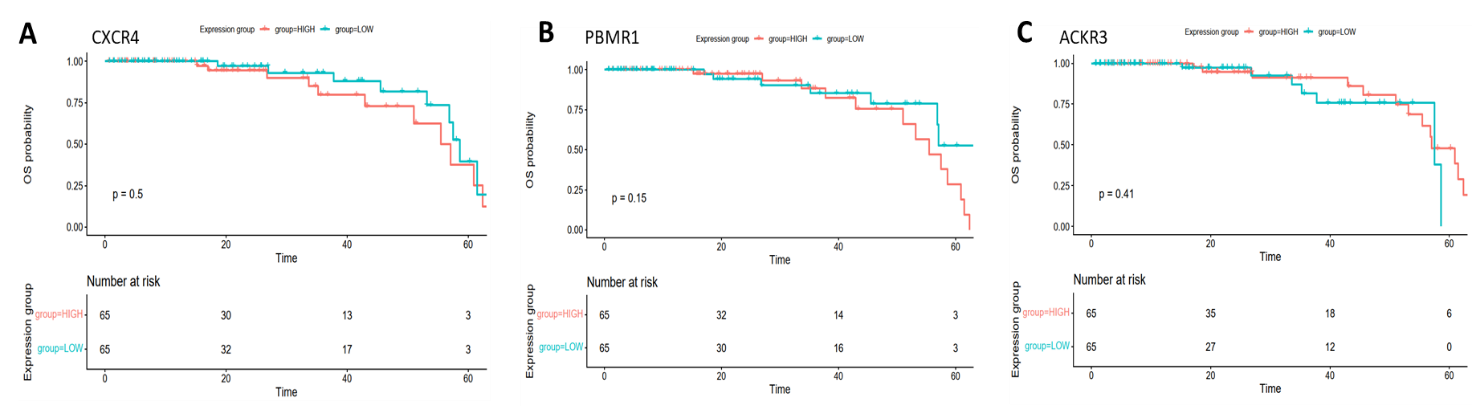


**Figure S7**. **Kaplan-Meier survival analysis for Everolimus treated Checkmate025 patients according to CXCR4, PBRM1, and CXCR7.** Overall survival (OS) analyses were performed within the everolimus-treated ccRCCs within publicly available CheckMate 025 (CM-025) trial to explore the clinical relevance of gene expression patterns. Gene expression–based stratification was performed classifying samples as high or low expression relative to the gene-specific median. Kaplan-Meier survival analysis for patients treated with Everolimus stratified by (A) CXCR4 (B) PBRM1and (C) CXCR7 expression. Kaplan–Meier survival curves were estimated for each comparison and evaluated using the log-rank test. Univariable Cox proportional hazards regression models were fitted to estimate hazard ratios (HRs) and 95% confidence intervals. Only comparisons with complete survival information and a minimum number of evaluable samples were considered.
