# Supplementary material for: CXCR4, CXCR7 and PBRM1 are responsible for everolimus and cabozantinib resistance in human renal cancer cells
Source: Cell Death Discov. 2026 Mar 28;12:202. doi: 10.1038/s41420-026-03026-w (PMC13149507; doi:10.1038/s41420-026-03026-w)
Supplement: Supplementary file 2 — Supplementary Materials and Methods [file 41420_2026_3026_MOESM2_ESM.docx]

**Supplementary Materials and Methods**

**Cell culture.** Human renal carcinoma cell lines SN12C were cultured under standard culture condition at 37 °C in a humidified atmosphere of 5% CO2. The RAD001-resistant SN12C cell lines (SN12C/RAD) were established by exposing the RAD001-sensitive SN12C parental cells to RAD001 from 1 nM to the final 20 μM over a 12-month period. Cells were cultured in Dulbecco's Modified Eagle Medium (Sigma) supplemented with 10% fetal bovine serum (FBS) and 1% penicillin and streptomycin (Invitrogen). Resistant cells were routinely cultured in complete medium containing relative concentration of RAD001 (1).

**Bioinformatic Analyses**

**Everolimus treated ccRCCs data source and cohort selection.**

Gene expression and clinical data were obtained from the publicly available supplementary material of Braun et al. (2), derived from the phase III CheckMate 025 (CM-025) trial. A total of 130 samples from the CM-025 everolimus arm met inclusion criteria and were included in the analyses. Expression data were available for all target genes. Data were extracted from the Excel file 41591_2020_839_MOESM2_ESM.xlsx, using the following sheets:

S1_Clinical_and_Immune_Data for clinical and immune-related variables

S4A_RNA_Expression for normalized bulk RNA-sequencing expression data

Analyses were restricted to patients enrolled in the CM-025 cohort and treated with everolimus. Only samples with a valid RNA identifier (RNA_ID) and corresponding gene expression data were included. Sample identifiers were harmonized to ensure correct matching between clinical annotations and expression profiles.

**Survival Analysis.** Transcriptomic and clinical data for Clear Cell Renal Cell Carcinoma were sourced from the The Cancer Genome Atlas (TCGA-KIRC) cohort or everolimus-treated CM-025 cohort to explore the clinical relevance of gene expression patterns.

Survival analyses were performed using the TCGEx portal (https://tcgex.iyte.edu.tr/), powered by the TCGAbiolinks and survival R packages for data processing for TCGA-KIRC and everolimus-treated from CM-025. Kaplan-Meier Survival Curves To assess the prognostic impact of individual genes, patients were stratified into "High" and "Low" expression groups based on the median mRNA expression of PBRM1, CXCR4, ACKR3, FOXP3, YY1, and MTOR. Overall Survival (OS) was compared between these groups using Kaplan-Meier estimator curves, and statistical significance was determined using the log-rank test. To evaluate the independent prognostic value of the selected targets, a Multivariable Cox Proportional Hazards regression was performed. The model included PBRM1, CXCR4, ACKR3, YY1, and FOXP3 as covariates to estimate Hazard Ratios (HR) with 95% Confidence Intervals (CI). The proportional hazards assumption was verified, and the global model significance was assessed via the log-rank test. A p-value < 0.05 was considered statistically significant.

**RNA Isolation and Real-Time Reverse Transcription-Polymerase Chain Reaction.**

RNA was extracted from cell lines with TRIzol Reagent (Invitrogen, Carlsbad, California, USA) following the manufacturer’s instructions. cDNA was synthesized using 200 ng RNA and 100 U Superscript III (Invitrogen, Karlsruhe, Germany) and random hexamer primers (Invitrogen) according to the manifacturer’s instructions. The gene-specific primers used for the amplification were as follows:

Cyclin D1: FW: 5’- GCTGCGAAGTGGAAACCATC -3’

RV: 5’- CCTCCTTCTGCACACATTTGAA -3’

VEGFA: FW: 5’- CCATCACCATCGACAGAACA -3’

RV: 5’- GGTGGGTGTGTCTACAGGAA -3’

**ImmunobloBlot.**

Cells were lysed in a whole-cell buffer containing protease and phosphatase (10 mM NaF, 10 mM Na-pyrophosphate, 1 mM Na_3_VO_4_) inhibitors. Anti-Axl (C89E7) Rabbit Monoclonal Antibody was obtained from Cell Signaling (Danvers, Massachusetts, USA), anti-PBRM1 Rabbit Polyclonal was obtained from Bethyl (Montgomery, TX, USA). Secondary antibody includes goat anti-rabbit-HRP (Jackson ImmunoResearch, West Grove, Pennsylvania, USA). The signal was revealed through chemoluminescence (Pierce ECL Western Blotting Substrate, Thermo Fisher Scientific). Protein expression was detected with Image Acquisition using iBright Imaging Systems (Invitrogen).

**References**

1. Ieranò C, Santagata S, Napolitano M, Guardia F, Grimaldi A, Antignani E, et al. CXCR4 and CXCR7 transduce through mTOR in human renal cancer cells. Cell Death Dis. 2014;5(7):e1310.

2. Braun DA, Hou Y, Bakouny Z, Ficial M, Sant' Angelo M, Forman J, et al. Interplay of somatic alterations and immune infiltration modulates response to PD-1 blockade in advanced clear cell renal cell carcinoma. Nat Med. 2020;26(6):909-18.
