# Supplementary material for: CXCR4, CXCR7 and PBRM1 are responsible for everolimus and cabozantinib resistance in human renal cancer cells
Source: Cell Death Discov. 2026 Mar 28;12:202. doi: 10.1038/s41420-026-03026-w (PMC13149507; doi:10.1038/s41420-026-03026-w)
Supplement: Supplementary file 3 — Uncropped Western blot [file 41420_2026_3026_MOESM3_ESM.docx]

1. **Uncropped Western blots for Figure 2A (iii)**

**
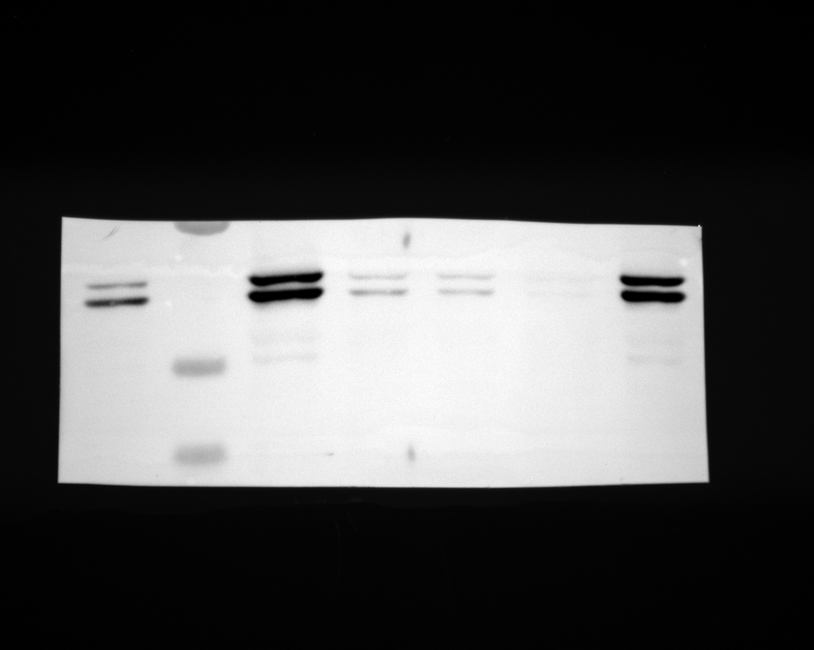
**

42-44 kDa -

**IB: pERK A498**

**
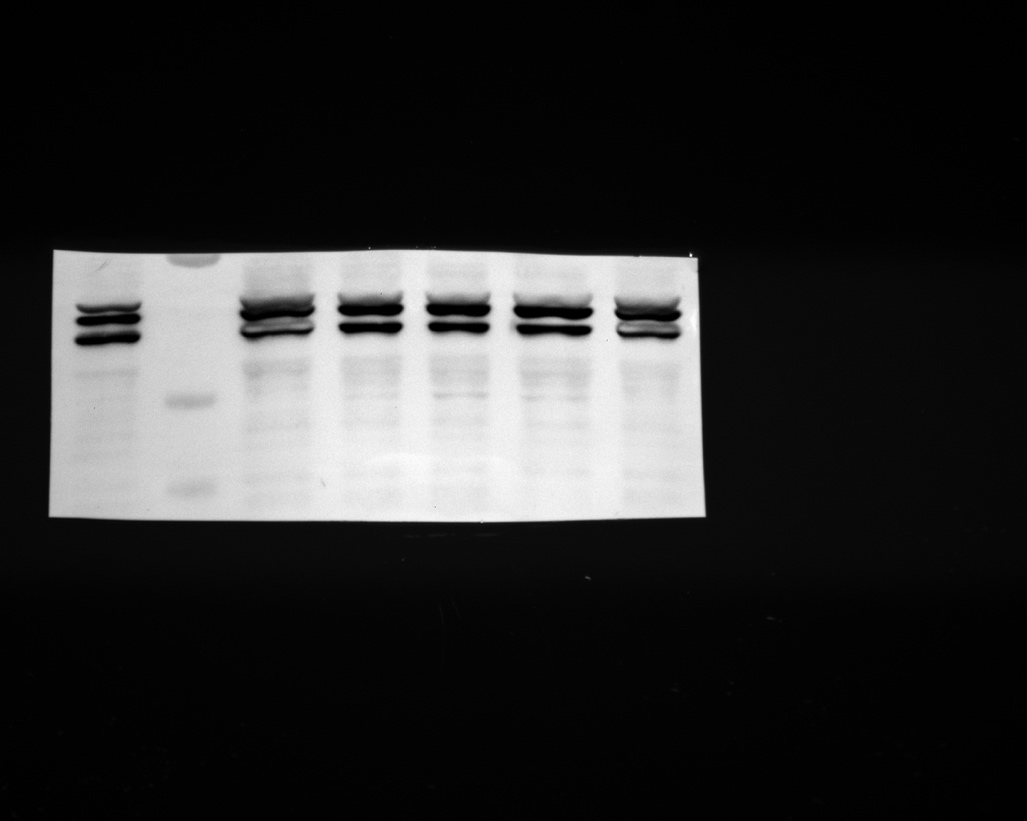
**

42-44 kDa -

**IB: ERK TOT A498**

**
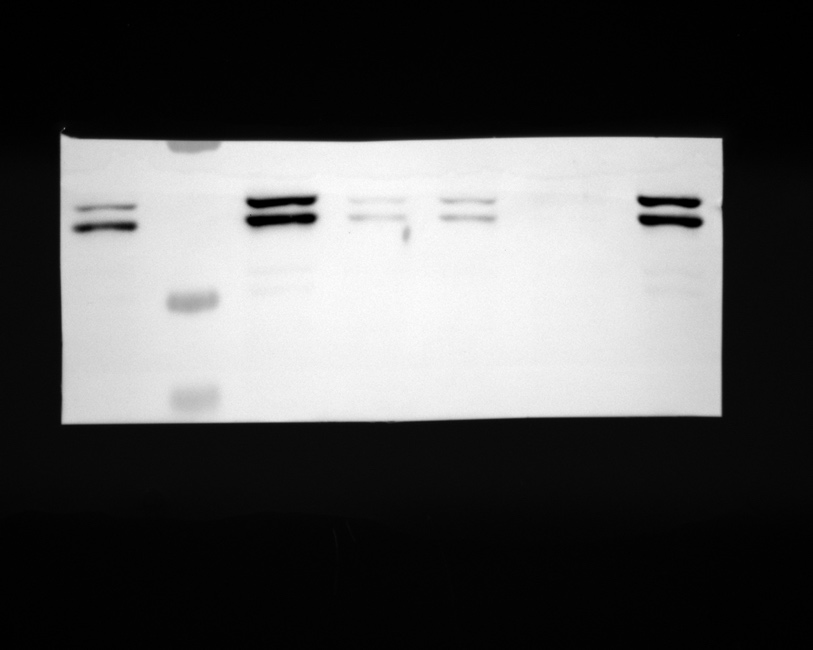
**

42-44 kDa -

**IB: pERK A498-RAD10**

**
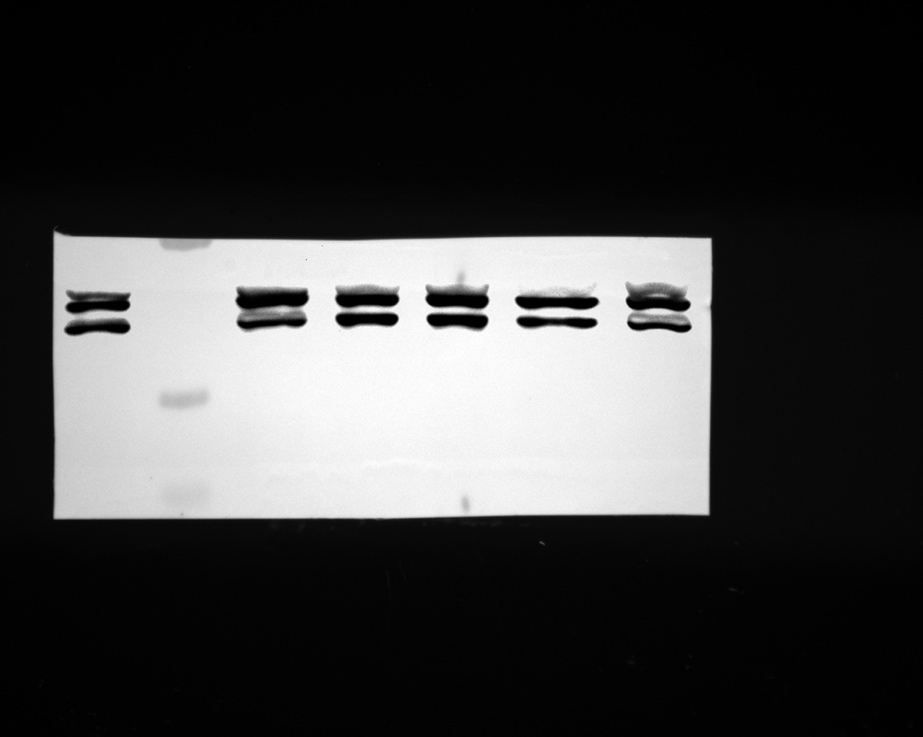
**

42-44 kDa -

**IB: ERK TOT A498-RAD10**

**
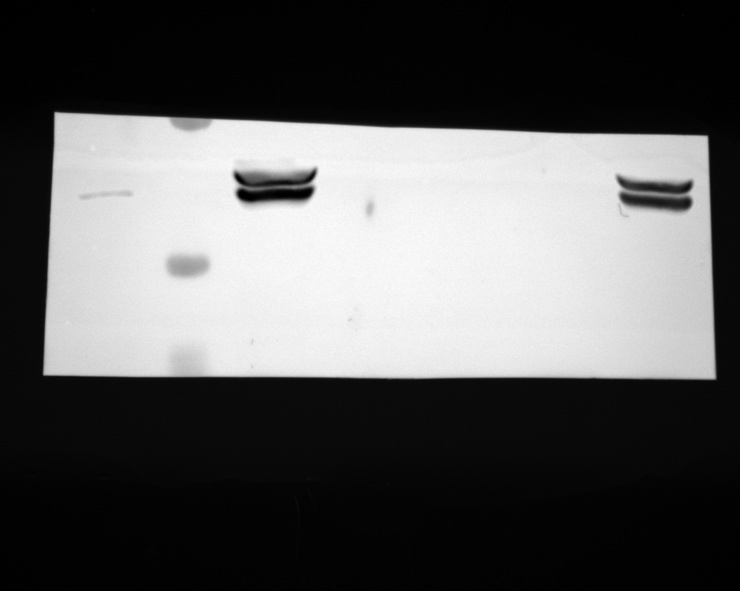
**

42-44 kDa -

**IB: pERK A498-RAD10shPBRM1**

**
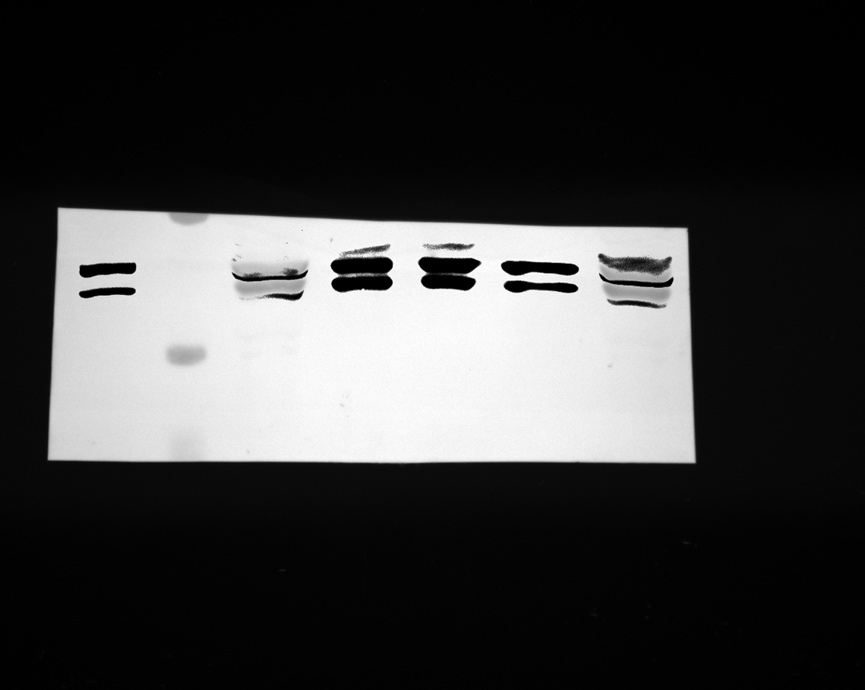
**

42-44 kDa -

**IB: ERK TOT A498-RAD10shPBRM1**

**
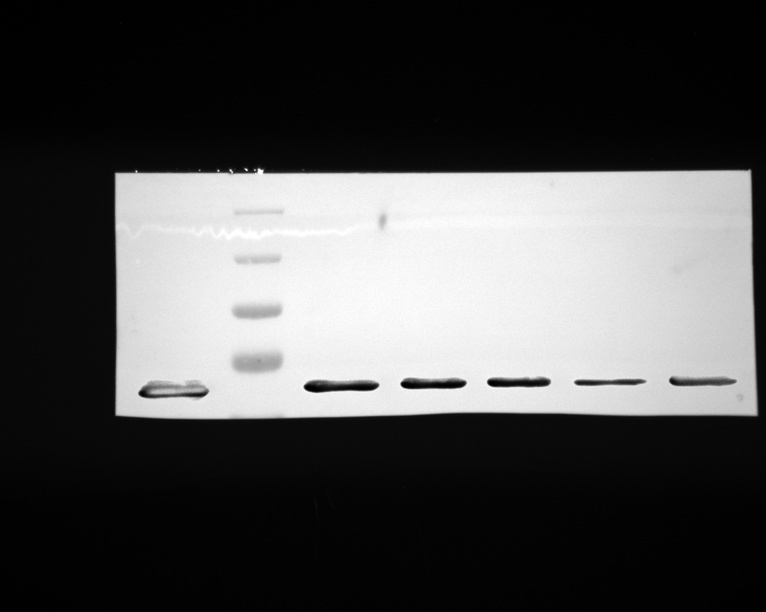
**

65 kDa -

**IB: YY1 A498**

**
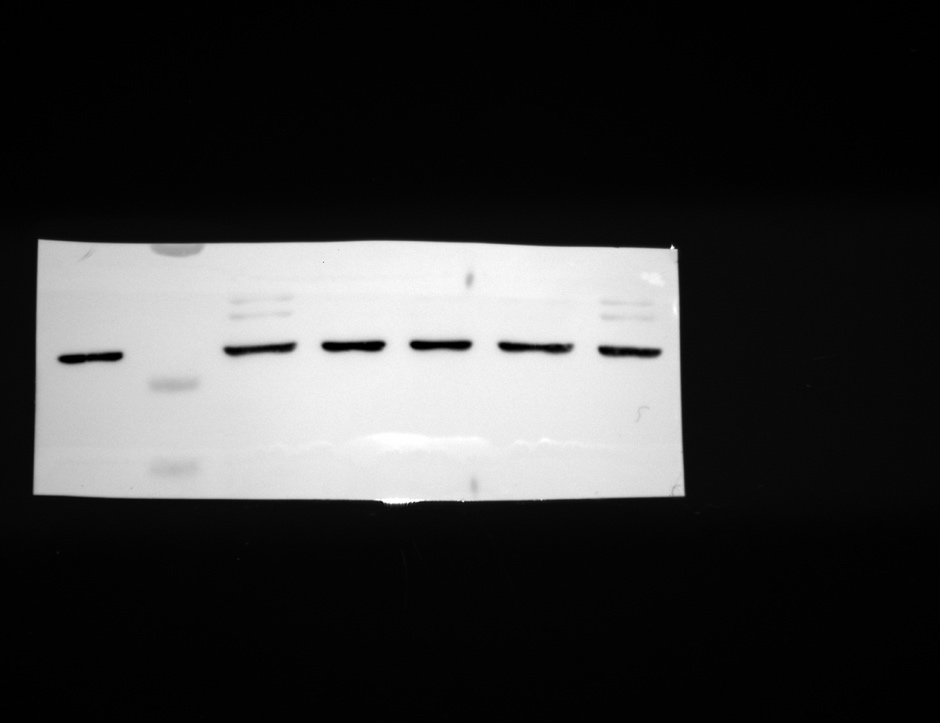
**

36 kDa -

**IB: GAPDH A498**

**
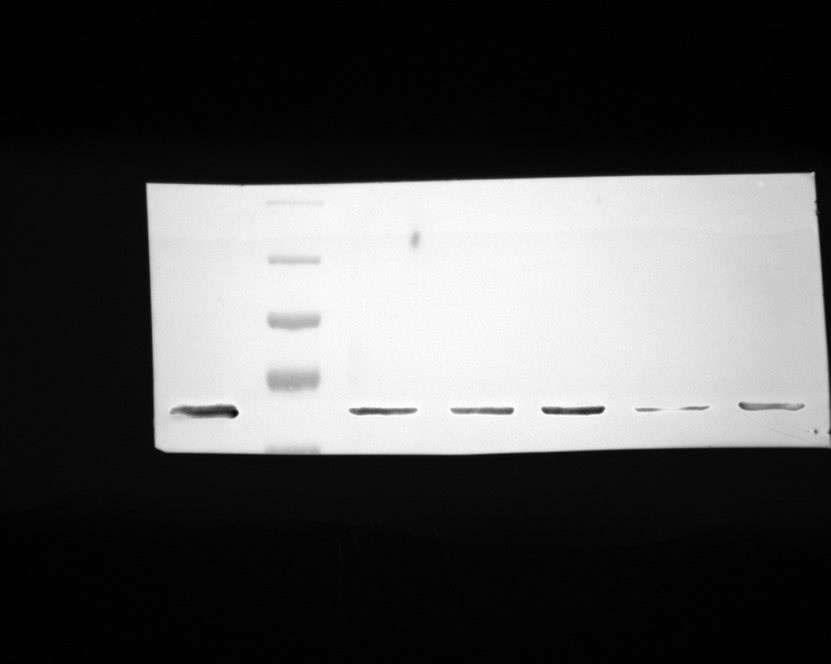
**

65 kDa -

**IB: YY1 A498-RAD10**

**
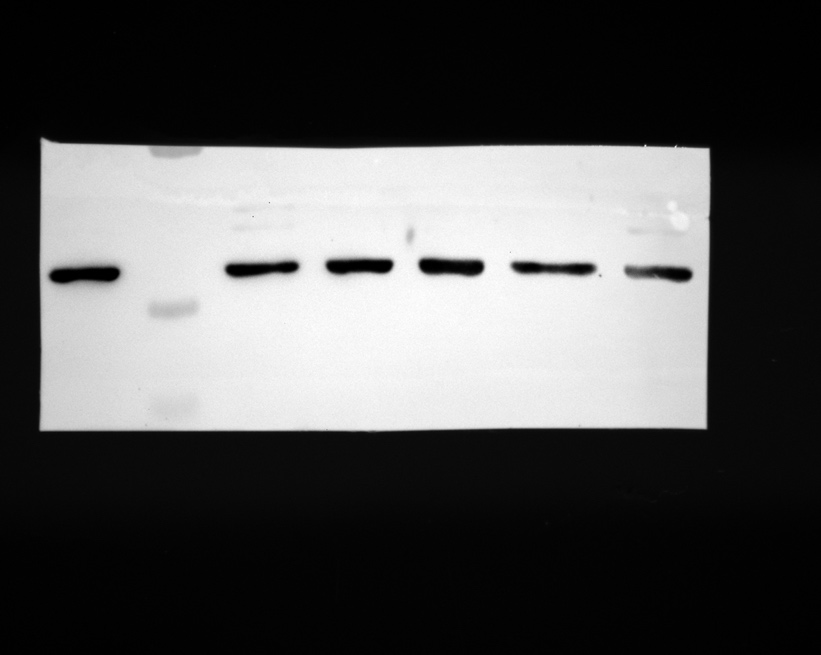
**

36 kDa -

**IB: GAPDH A498-RAD10**

**
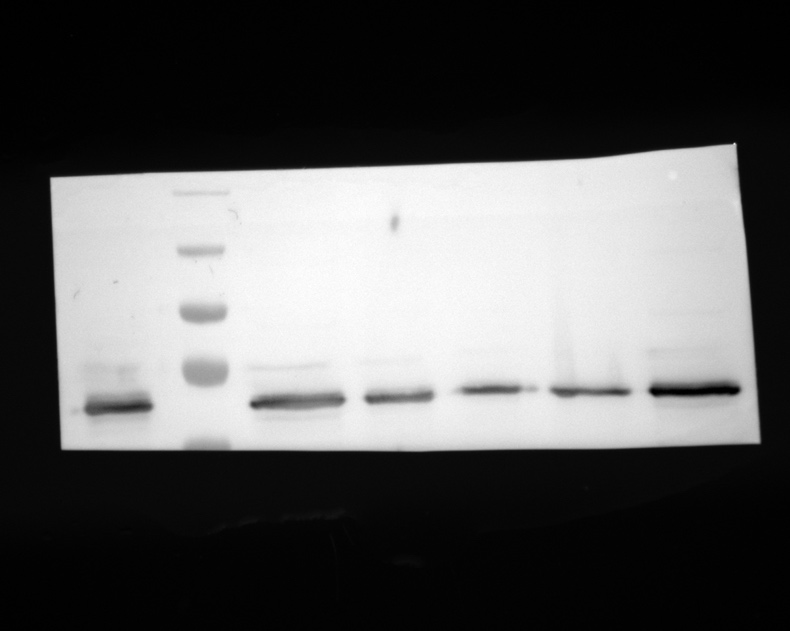
**

65 kDa -

**IB: YY1 A498-RAD10shPBRM1**

**
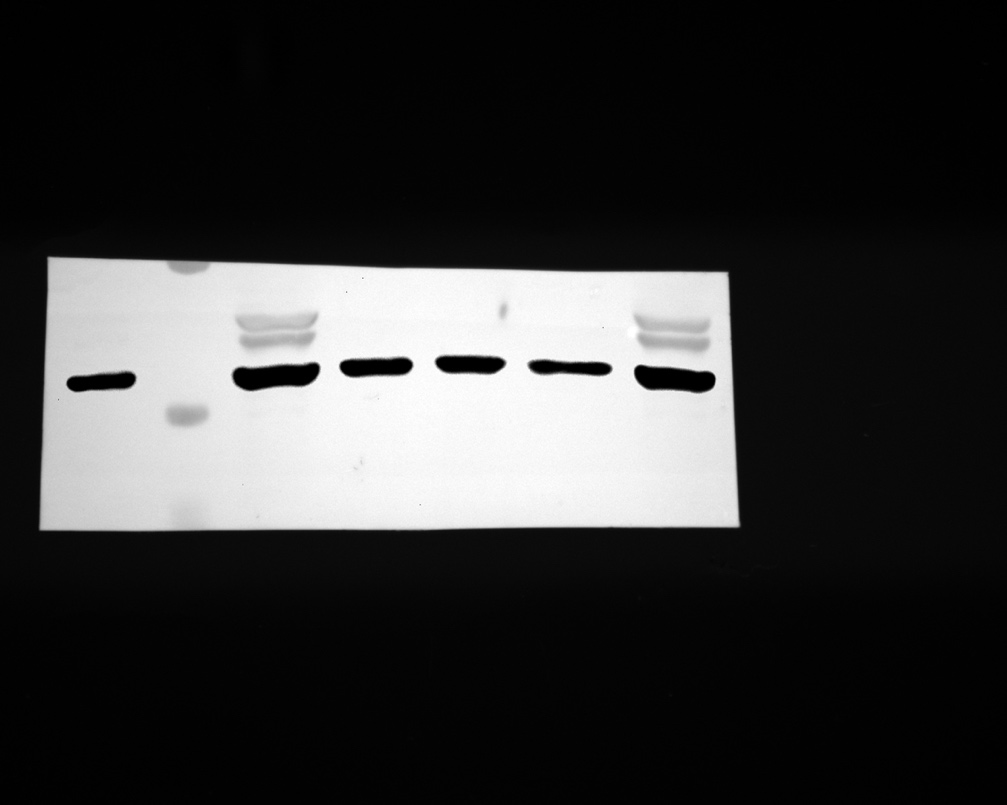
**

36 kDa -

**IB: GAPDH A498-RAD10shPBRM1**

1. **Uncropped Western blots for Fig. 3C**

**
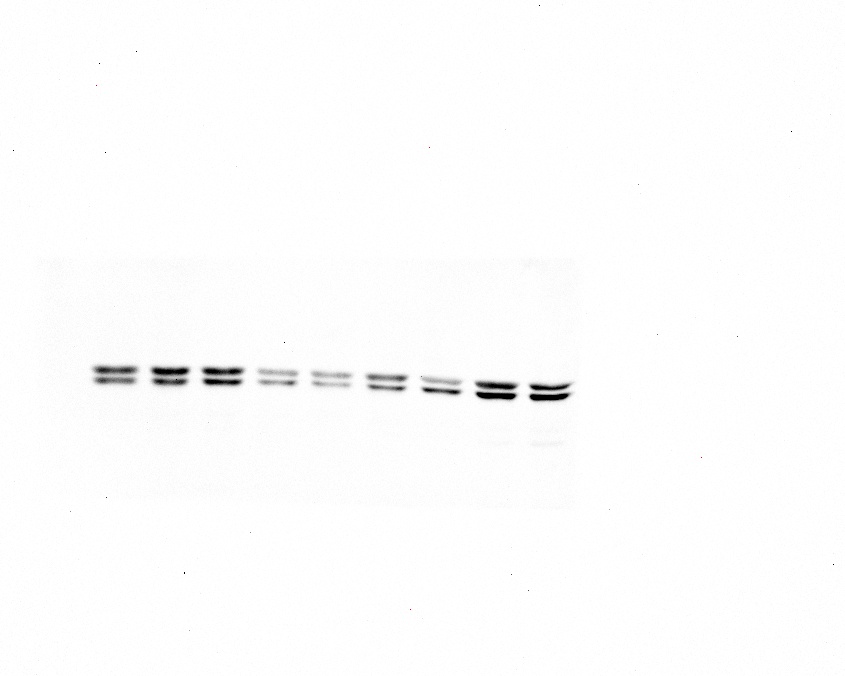
**

42-44 kDa -

**IB: pERK**

**
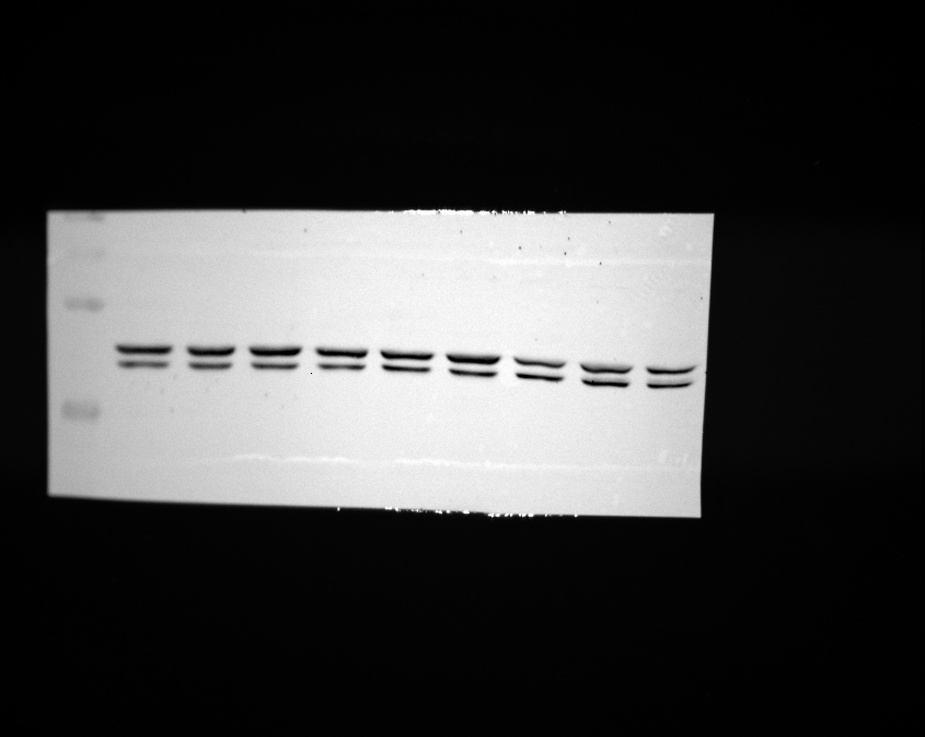
**

42-44 kDa -

**IB: ERK**

1. **Uncropped Western blots for Fig. 3D**

**
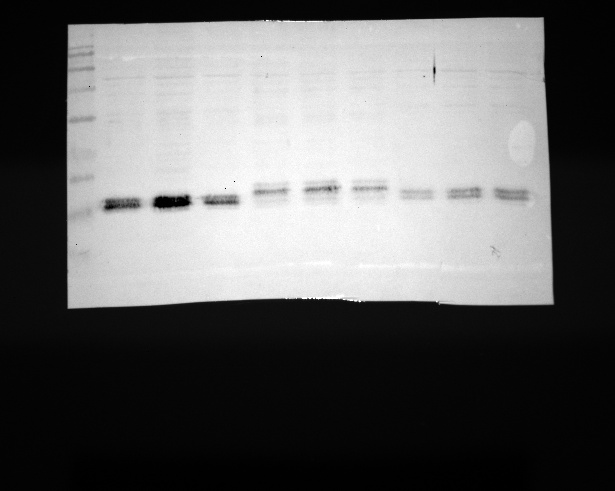
**

15 kDa -

**IB: p4EBP1**

**
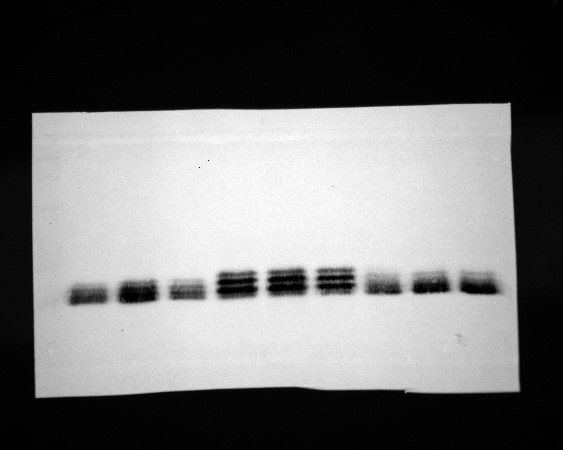
**

15 kDa -

**IB: 4EBP1**

1. **Uncropped Western blots for Fig. 3E**


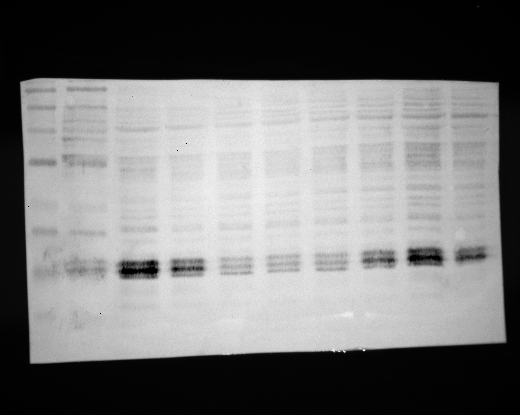


15 kDa -

**IB: p4EBP1**

**
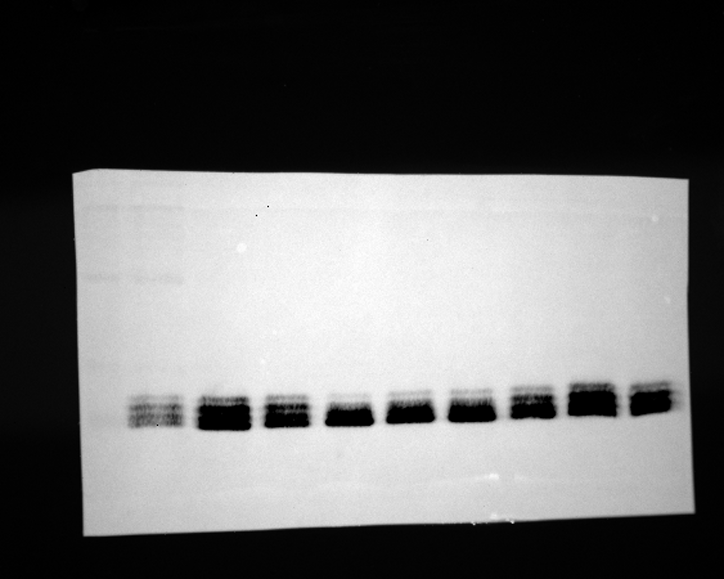
**

15 kDa -

**IB: 4EBP1**

1. **Uncropped Western blots for Fig. 5C**

**
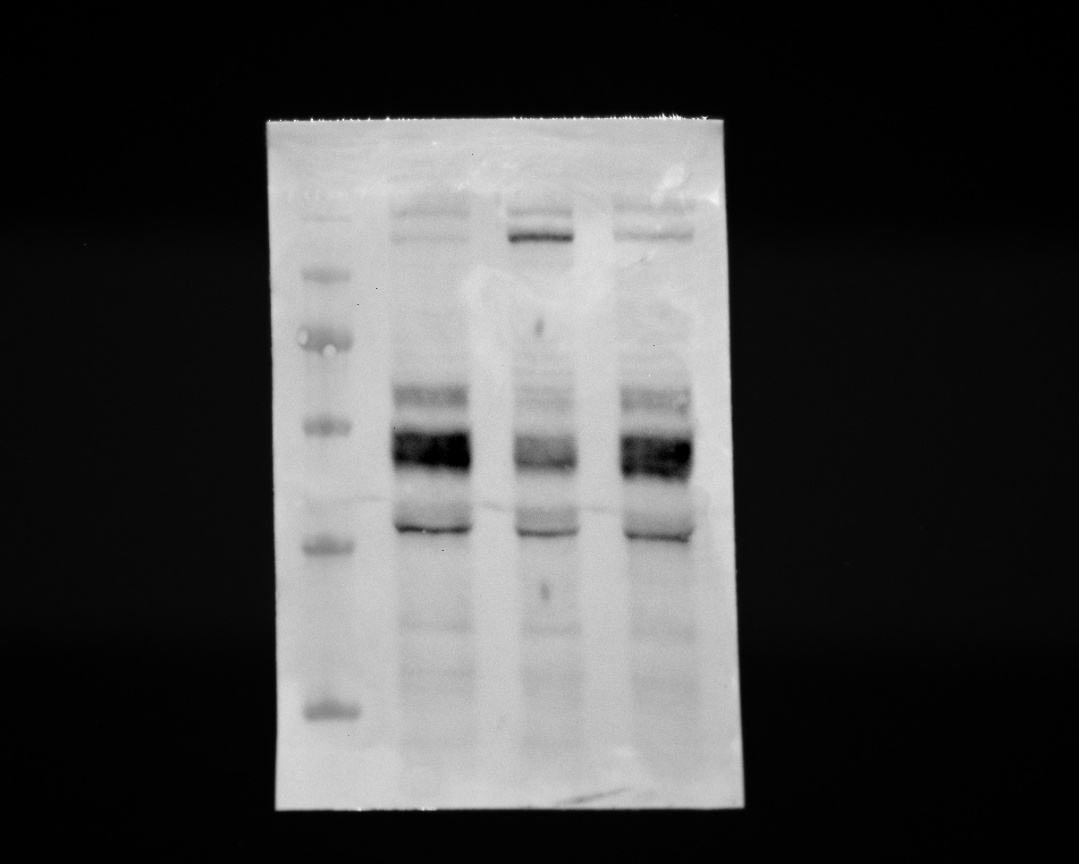
**

145 kDa -

**IB: pMET**

**
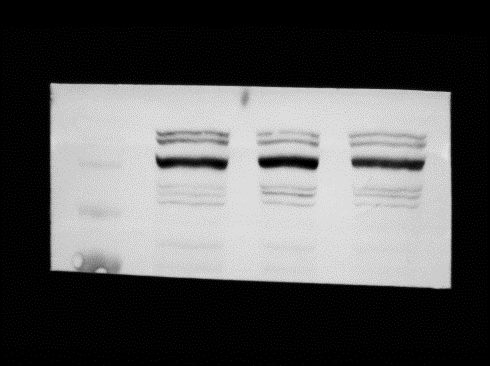
**

145 kDa -

**IB: MET**

**
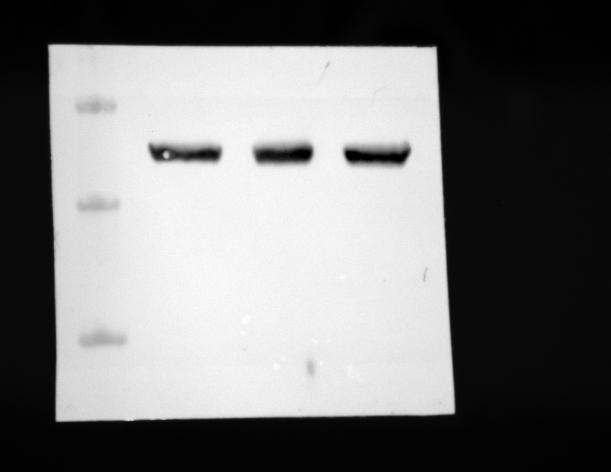
**

55 kDa -

**IB: TUBULIN**
